# Role of LGR5-positive mesenchymal cells in craniofacial development

**DOI:** 10.3389/fcell.2022.810527

**Published:** 2022-09-05

**Authors:** Kristýna Olbertová, Dušan Hrčkulák, Vítězslav Kříž, Wojciech Jesionek, Jan Kubovčiak, Milan Ešner, Vladimír Kořínek, Marcela Buchtová

**Affiliations:** ^1^ Laboratory of Molecular Morphogenesis, Institute of Animal Physiology and Genetics, Czech Academy of Sciences, Brno, Czechia; ^2^ Department of Experimental Biology, Faculty of Science, Masaryk University, Brno, Czechia; ^3^ Laboratory of Cell and Developmental Biology, Institute of Molecular Genetics, Czech Academy of Sciences, Prague, Czechia; ^4^ Cellular Imaging Core Facility, Central European Institute of Technology (CEITEC), Masaryk University, Brno, Czechia; ^5^ Laboratory of Genomics and Bioinformatics, Institute of Molecular Genetics, Czech Academy of Sciences, Prague, Czechia

**Keywords:** LGR5, tongue, palate, vomeronasal organ, craniofacial, stem cell, epithelial folding

## Abstract

Leucine Rich Repeat Containing G Protein-Coupled Receptor 5 (LGR5), a Wnt pathway member, has been previously recognised as a stem cell marker in numerous epithelial tissues. In this study, we used *Lgr5-EGFP-CreERT2* mice to analyse the distribution of LGR5-positive cells during craniofacial development. LGR5 expressing cells were primarily located in the mesenchyme adjacent to the craniofacial epithelial structures undergoing folding, such as the nasopharyngeal duct, lingual groove, and vomeronasal organ. To follow the fate of LGR5-positive cells, we performed lineage tracing using an inducible Cre knock-in allele in combination with Rosa26-tdTomato reporter mice. The slight expansion of LGR5-positive cells was found around the vomeronasal organ, in the nasal cavity, and around the epithelium in the lingual groove. However, most LGR5 expressing cells remained in their original location, possibly supporting their signalling function for adjacent epithelium rather than exerting their role as progenitor cells for the craniofacial structures. Moreover, *Lgr5* knockout mice displayed distinct defects in LGR5-positive areas, especially in the reduction of the nasopharyngeal duct, the alteration of the palatal shelves shape, abnormal epithelial folding in the lingual groove area, and the disruption of salivary gland development. The latter defect manifested as an atypical number and localisation of the glandular ducts. The gene expression of several Wnt pathway members (*Rspo1-3, Axin2*) was altered in *Lgr5*-deficient animals. However, the difference was not found in sorted EGFP-positive cells obtained from *Lgr5* ^*+/+*^ and *Lgr5* ^*−/−*^ animals*.* Expression profiling of LGR5-positive cells revealed the expression of several markers of mesenchymal cells, antagonists, as well as agonists, of Wnt signalling, and molecules associated with the basal membrane. Therefore, LGR5-positive cells in the craniofacial area represent a very specific population of mesenchymal cells adjacent to the epithelium undergoing folding or groove formation. Our results indicate a possible novel role of LGR5 in the regulation of morphogenetic processes during the formation of complex epithelial structures in the craniofacial areas, a role which is not related to the stem cell properties of LGR5-positive cells as was previously defined for various epithelial tissues.

## Introduction

Leucine Rich Repeat Containing G Protein-Coupled Receptor 5 (LGR5), also known as a GPR49, is a member of the large LGR protein family comprising the seven-span transmembrane receptors. LGR5 and the related LGR4 and LGR6 proteins represent the B subgroup of the family that potentiates Wnt signalling (reviewed by Kriz and Korinek, 2018). All three LGRs interact with the secreted Wnt pathway agonists R-spondins (RSPOs) (Carmon et al., 2011; de Lau et al., 2011; Glinka et al., 2011). RSPO binding to LGRs leads to the clearance of cell surface transmembrane E3 ubiquitin ligases Ring Finger 43 (RNF43) and Zinc And Ring Finger 3 (ZNRF3). The ligases function as Wnt pathway antagonists by promoting the turnover of the Wnt ligand-receptor complex composed of the Frizzled (FZD) receptor and Low-Density Lipoprotein Receptor-Related Protein 6 (LRP6) co-receptor. The RNF43/ZNRF3 activity is suppressed by the internalisation of the RSPO–LGR4/5–RNF43/ZNRF3 complex formed upon RSPO binding to LGR (Hao et al., 2012). The RSPO-LGR interaction plays an important role in various biological processes such as the development of limbs, lungs, nails, placenta and odontogenesis (Nagano, 2019). Moreover, RSPO-LGR signalling is essential for adult stem and progenitor cell maintenance and proliferation (reviewed in Barker et al., 2013).

LGR5 is widely used as a marker of stem cells in many adult tissues, including the epithelia of the gastrointestinal tracts or hair follicles (reviewed in Barker and Clevers, 2010). During early craniofacial development, *Lgr5* mRNA (under the older name *Fex*) was observed in the mesenchyme surrounding the mandibular cleft and the most lateral aspects of the tongue at embryonic day (E) 10.5, E12.5, and E13.5 (Hermey et al., 1999). At later stages, LGR5-positive cells were found in the otic and optic vesicle and in migrating neural crest cells in the craniofacial region (Boddupally et al., 2016). Immunohistochemistry was used to detect LGR5 at E14.5 in the tongue epithelium and mandibular mesenchyme (Morita et al., 2004). Moreover, LGR5 is produced at the bottom of the circumvallate taste papilla and in the area adjacent to the opening of the duct of Von Ebner´s gland, where stem/progenitor cells are localised (Yee et al., 2013). Nevertheless, a detailed *Lgr5* expression pattern during later craniofacial organogenesis (focusing on individual structures) has not been analysed yet.

In this study, we used transgenic mice *Lgr5-EGFP-IRES-CreERT2* to detect LGR5-positive cells in craniofacial structures in embryonic and early postnatal stages. As large subpopulations of LGR5-positive cells were located in specific areas of the craniofacial mesenchyme, we asked whether these cells represent stem/progenitor cells and contribute to cell renewal of craniofacial structures. To target this point, we employed *Rosa26-tdTomato* “reporter” mice to perform the fate mapping of LGR5-positive cells through development. Next, we analysed the developmental defects in the craniofacial areas of *Lgr5*-deficient mice and the changes in Wnt signalling in these animals. Finally, we performed transcriptomic analyses of *Lgr5*-positive cells to uncover these cells’ gene expression profiles and possible involvement in signalling pathways other than the canonical Wnt signalling.

## Materials and methods

### Mouse strains, genotyping, and LGR5-positive cell lineage tracing

The housing of mice and *in vivo* experiments were performed in compliance with the European Communities Council Directive of 24 November 1986 (86/609/EEC) and national and institutional guidelines. Animal care and experimental procedures were approved by the Animal Care Committee of the Institute of Molecular Genetics (Ref. 58/2017). *Lgr5-EGFP-IRES-CreERT2* mice were obtained from the Jackson Laboratory (Bar Harbor, United States; strain: *Lgr5* ^*tm1(cre/ERT2)Cle*^/J). The mice harbour the *Lgr5-EGFP-IRES-CreERT2* “knock-in” allele, which disrupts *Lgr5* gene function and drives the expression of EGFP and CreERT protein from the endogenous *Lgr5* locus. The design of knock-in *Lgr5-EGFP-IRES-CreERT2* mice expressing a tamoxifen-regulated variant of Cre recombinase from the *Lgr5* locus, including the efficiency of Cre-mediated recombination, was described previously (Barker et al., 2007).

Animals were genotyped by PCR using a set of three primers: *Lgr5*-specific reverse primer detecting the wild-type (WT) allele 5′ GAC​GTC​TGG​TGA​GCT​GCA​GAA​G 3′, EGFP-specific reverse primer detecting the knock-in allele 5′ GAA​GAA​GTC​GTG​CTG​CTT​CAT​GT 3′, and the common forward primer 5′ GAC​GTC​TGG​TGA​GCT​GCA​GAA​G 3′ annealing to the 5′untranslated region (UTR) of the *Lgr5* gene. PCR (40 cycles; denaturation: 95°C, 30 s; annealing: 63°C, 30 s; extension: 72°C, 30 s) on genomic DNA isolated from the tail tips of the heterozygous animals produced a 450 bp band corresponding to the WT allele and 360 bp band corresponding to the knock-in allele. We analysed at least five mice embryos for each embryonic stage. To analyse the fate of LGR5-positive cells, *Lgr5-EGFP-IRES-CreERT2* mice were crossed with homozygous Rosa26-tdTomato “reporter” mice [Jackson Laboratory, strain: 129S6-*Gt(ROSA)26Sor* ^*tm14(CAG-tdTomato)Hze*^/J mice] (Madisen et al., 2010). *Rosa26-tdTomato* animals produce an mRNA encoding tandem dimer of red fluorescent Tomato (tdTomato) protein from the ubiquitously active *Rosa26* locus. The mRNA is not translated (and the tdTomato protein produced) unless a transcriptional stop signal flanked by a pair of *loxP* sites is removed from the genome by Cre-mediated excision. The Cre activity in embryos at E12.5 and E13.5 was induced by a single dose of tamoxifen (2 mg) in corn oil that was gavaged into the pregnant females. The mice were sacrificed at several time points (E15.5, E16.5, E18.5) after the Cre activation. We analysed three to five mice embryos at each time point.

### Immunohistochemistry and native fluorescence imaging

Embryos were collected at different stages of embryonal development (E11.5, E12.5, E13.5, E14.5, E16.5, E18.5, P4). They were euthanised by decapitation and fixed in 4–10% paraformaldehyde (cat. No. 23700-31000, PFA; Penta Chemicals Unlimited, Czech Republic) overnight. Specimens for histological and immunohistochemical analysis were decalcified in 10% EDTA in 4% PFA at room temperature (RT) and then embedded in paraffin. Paraffin-embedded tissues were cut along the transverse plane to get serial transversal histological sections. The sections were divided into two groups. One was stained with Hematoxylin-Eosin solution, while the neighbouring slides were used for immunohistochemical analysis. Antigen retrieval was performed in the steam water bath (97°C) in DAKO Target Retrieval Solution, pH 9 (Dako Agilent, United States) for 15–30 min. To prevent the unspecific binding of antibodies, blocking serum was applied to the samples for 30 min. Slides were incubated with the primary antibody anti-EGFP (chicken polyclonal, cat. No. ab13970; Abcam, United Kingdom; dilution 1:200), anti-vimentin (D21H3) XP (rabbit monoclonal, cat. No. 5741; Cell Signaling, United States, dilution 1:100), anti-RFP (rabbit polyclonal, cat. No. 600-401-379; Thermo Fisher Scientific, United States; dilution 1:200), anti-β-catenin XP (rabbit monoclonal, cat. No. 8480; Cell Signaling, United States, dilution 1:100)), anti-laminin (rabbit polyclonal, cat. No. Z0097; Dako, Denmark, dilution 1:100), and anti-E-cadherin (rabbit polyclonal, cat. No. ab15148; Abcam, United Kingdom, dilution 1:30) for 1 h or (up to) overnight. The secondary antibodies (anti-chicken Alexa Fluor^®^ 488, cat. No. A11039, Life Technologies, United States; dilution 1:200 and anti-rabbit AlexaFluor^®^ 555, cat. No. A31572, Life Technologies, United States, dilution 1:200) were applied for 30 min and 4′,6-diamidino-2-phenylindole (DAPI; 1 μg/ml; cat. No. D3571, Invitrogen, United States) was used for the counterstaining. Photos were taken using the fluorescence microscope Leica DM LB2 (Leica Microsystems, Germany). Pictures were processed by Adobe Photoshop (Adobe Systems Incorporated, United States). For three-dimensional (3D)/whole-mount imaging of native EGFP and/or tdTomato fluorescence, maxillary regions were surgically excised and fixed in 4% PFA pH 7.4 overnight at 4°C. Specimens were washed with phosphate-buffered saline (PBS) and counterstained with DAPI for 2 h at 25°C. Fluorescent 3D-stack images were taken under a spinning-disc microscope Dragonfly (Andor, Belfast, United Kingdom) using a 20× (water immersion) objective; 3D stacks, maximal projections, and 3D videos were processed in IMARIS software.

### Quantification of LGR5-positive cells

The number of LGR5-positive cells on fluorescently labelled tissue sections were determined using Cell Profiler 4.2.1—an open-source software (Stirling et al., 2021). At least four sections of nasopharyngeal duct area at each analyzed stage (E11.5, E12.5, E13.5, E14.5, E18.5) were analysed. Nuclei were detected in the DAPI channel using a minimum cross-entropy segmentation algorithm. Nuclei outlines were expanded by 15 pixels to partially cover the cytoplasm of the cells. In this region, we measured the intensity of the LGR5 staining. The threshold for the LGR5 positivity was based on the mean intensity, and the minimum value was set as 0.1. The number of positive cells was automatically counted for all images. Control images with depicted outlines of LGR5 cells were exported for visual control of segmentation (Figure 2Q). The percentage of LGR5-positive cells was calculated as a ratio between the total number of DAPI nuclei and the number of LGR5-positive cells.

### Analyses of signal colocalisation

Images were analysed using ZEN Blue software (Carl Zeiss Microscopy GmbH, Germany), version 3.2. Separate TIFF images for each channel were imported into ZEN software, and corresponding channels were overlaid. All analyses were performed only on pixels above the threshold. An individual threshold was set for each channel and image. To quantify colocalisation, we used the colocalisation coefficient defined by the following formula:

$$\frac{\sum PixelsCh_{1,Colocalized}}{\sum PixelsCh_{1,Total}}$$

This coefficient indicates the relative number of colocalised pixels in each channel in relation to the total number of pixels above the selected threshold. Coefficient 1 is for red color, coefficient 2 for green color. Coefficients have values between 0 and 1. The higher number is corresponding to higher proportion of selected pixels in channel one colocalising with pixels in channel two. We also calculated area of signal in red and green channel and normalized it to DAPI channel. The optimal threshold for the DAPI channel was found to cover most of the nuclei of the image, and the number of pixels above this threshold was counted. The number of pixels in red and green channels was normalised to this number to obtain the relative area on the image stained by the red or green channel.

### Colocalisation analyses in 3D

Colocalisation analysis of individual structures on the 3D visualisation of the maxillary region was performed as follows. Each structure was 3D cropped from the complete Z-stack using IMARIS graphical software to limit the influence of surrounding cells. The resulting sections were loaded to ImageJ software (Schneider et al., 2012) and analysed by the JACoP plugin (Bolte and Cordelieres 2006) after setting the thresholds for both channels (tdTomato and EGFP) to limit background signals and cover most of the cell-specific signals. The resulting Mander’s coefficient 1 shows tdTomato signal overlapping the EGFP signal, and Mander’s coefficient 2 shows the EGFP signal overlapping the tdTomato signal.

### Messenger RNA detection

The expression of *Lgr5, Axin2, Rspo1, Rspo2,* and *Rspo3* was analysed by RNAscope (Advanced Cell Diagnostic, United States) in mice at E16.5 or E18.5. We analysed at least five mice embryos for each stage. Mice embryos were euthanised by decapitation and fixed in 4–10% PFA for a maximum of 48 h. After fixation, samples were decalcified in 10% EDTA for 3 days at 4°C, dehydrated by standard ethanol series followed by xylene. The samples were embedded in paraffin and sectioned at 5 µm. Tissues were processed using the RNAscope Multiplex Fluorescent v2 assay (cat. No. 323 110, Advanced Cell Diagnostics, United States). Samples were incubated in hydrogen peroxide (cat. No. 322 335, Advanced Cell Diagnostics, United States) at RT for 10 min before they were boiled in the target retrieval (cat. No. 322 001, Advanced Cell Diagnostics, United States) and pretreated with Protease Plus (cat. No. 322 331, Advanced Cell Diagnostic, United States). An LGR5 probe (RNAscope^®^ Probe Mm-LGR5, cat. No. 312171, Advanced Cell Diagnostics, United States) was used to detect transcripts, and DAPI (cat. No. 323 108, Advanced Cell Diagnostics, United States) was used for nucleus staining. Pictures were taken under a Leica DM LB2 microscope (Leica Microsystems, Germany) and processed by Adobe Photoshop 7.0.

### Flow cytometry and quantitative RT-PCR (qRT-PCR)

Mice embryos were collected at E16.5 (15 mice embryos). The embryos were decapitated, and the heads were divided into upper and lower jaws. The nasopharyngeal duct and palate areas were collected from the upper jaws, whereas the lingual grooves were isolated from the lower jaws. The isolated areas of each head were collected separately and cut into small pieces, and transferred to 24-well plates with 2.5 ml Collagenase P (3 U/mL; Colla-RO, col. No. 10103578001, Roche, United States) dissolved in Hanks’ balanced salts (HBBS, cat. No. 88284, Thermo Fisher Scientific, United States) and incubated for at least 1 hour at 37°C. During the enzymatic digestion, tissue pieces were gently pipetted up and down four times using a 1 ml pipette. After incubation, the cell suspension was centrifuged for 5 min at 600 x g. The supernatant was removed, and the pellet was resuspended in 500–1000 ml HBBS.

Cell suspensions were filtrated through a 70 µm sieve and stained for cell viability with Hoechst 33258 (cat. No. H3569, Thermo Fisher Scientific, United States). Cells were analysed by flow cytometry using a FACSAria IIu cell sorter (BD Biosciences, United States) and an Influx high-speed cell sorter (BD Biosciences, United States). Viable single-cell EGFP-positive or EGFP-negative populations were sorted into RNA lysis buffer (Qiagen, Germany). RNA was isolated from the sorted cells using an RNAeasy Micro kit according to the manufacturer’s protocol and included DNAse treatment (cat. No. 74004, Qiagen, Germany) and reverse transcription using MAXIMA reverse transcriptase (cat. No. EP0743, Thermo Fisher Scientific, United States). The LightCycler 480 apparatus and SYBR Green I Master Mix (cat. No. 04707516001, Roche Applied Science, Germany) were employed for qRT-PCR. Primers used for analysis are listed in Table 1. Cycle threshold (Ct) values were normalised to *beta-Actin* (*Actb*), and *Ubiquitin B* (*Ubb*) was used as a second housekeeping gene.

**TABLE 1:** List of primers for QPCR analyses.

Primers	Sequence
F-Actb	GGC​ATC​CTC​ACC​CTG​AAG​TA
R-Actb	AGG​TGT​GGT​GCC​AGA​TTT​TC
F-Axin2	TAG​GCG​GAA​TGA​AGA​TGG​AC
R-Axin2	CTG​GTC​ACC​CAA​CAA​GGA​GT
F-Nkd1	AGG​ACG​ACT​TCC​CCC​TAG​AA
R-Nkd1	TGC​AGC​AAG​CTG​GTA​ATG​TC
F-Sfrp2	CTT​CCC​CTG​GCC​AGA​CAT​G
R-Sfrp2	GGG​TTT​CCA​TGA​TGT​CGT​TGT
F-Ubb	ATG​TGA​AGG​CCA​AGA​TCC​AG
R-Ubb	TAA​TAG​CCA​CCC​CTC​AGA​CG

### Bulk RNA sequencing of LGR5-positive cells

RNA isolated from EGFP-positive and EGFP-negative cells obtained from the nasopharyngeal duct roof of heterozygotic animals at E16.5 were used for bulk RNA sequencing **(**RNA-seq). Sequencing libraries were prepared from total RNA using the Smarter Stranded Total RNA-seq Kit v2 Pico Input Mammalian (Takara, Japan), followed by size distribution analysis in the Agilent 2100 Bioanalyzer using a High Sensitivity DNA Kit (cat. No. 5067-4626, Agilent, United States). Libraries were sequenced in the Illumina NextSeq 500 instrument (Illumina, United States) using a 76 bp single-end configuration. Read quality was assessed using FastQC (Babraham Bioinformatics, Babraham Institute, United Kingdom). The nf-core/rnaseq bioinformatics pipeline version 3.5 (Ewels et al., 2020) was used for subsequent read processing. Individual steps included the removal of sequencing adapters and low-quality reads with Trim Galore (Babraham Bioinformatics, United Kingdom), mapping to the reference GRCm39 genome (Howe et al., 2021) using STAR, and quantification of gene expression with Salmon (Patro et al., 2017) using GRCh39 as a reference. The expression per gene served as input for differential expression analysis using the DESeq2 R Bioconductor package (Love et al., 2014). Genes not exhibiting expression of at least ten fragments in all samples were screened out before analysis. We created an experimental model in which the sample group was assumed as the main effect. Genes that had a minimum absolute log2 fold change of 1 (|log2| FC ≥ 1) and statistical significance (adjusted *p*-value < 0.05) between the compared sample groups were considered differentially expressed.

## Results

LGR5 is recognised as one of the most prominent markers of adult epithelial stem/progenitor cells (review Koo and Clevers 2014). Interestingly, in the craniofacial area, LGR5 expression is not restricted just to the epithelium (Nihara et al., 2021). Therefore, we mainly focused on LGR5-positive mesenchymal cells, their fate and their possible role in craniofacial morphogenesis. Due to the absence of reliable antibodies, staining of the EGFP protein was used to detect LGR5-producing cells in *Lgr5-EGFP-IRES-CreERT2* transgenic mice. However, we also performed *in situ Lgr5* mRNA detection on selected samples to verify our approach. This staining confirmed the suitability of using EGFP expression as a surrogate marker for LGR5-positive cells (Supplementary Figure S1).

### LGR5-positive cells are located in the palatal mesenchyme and nasopharyngeal area

**FIGURE 1:**
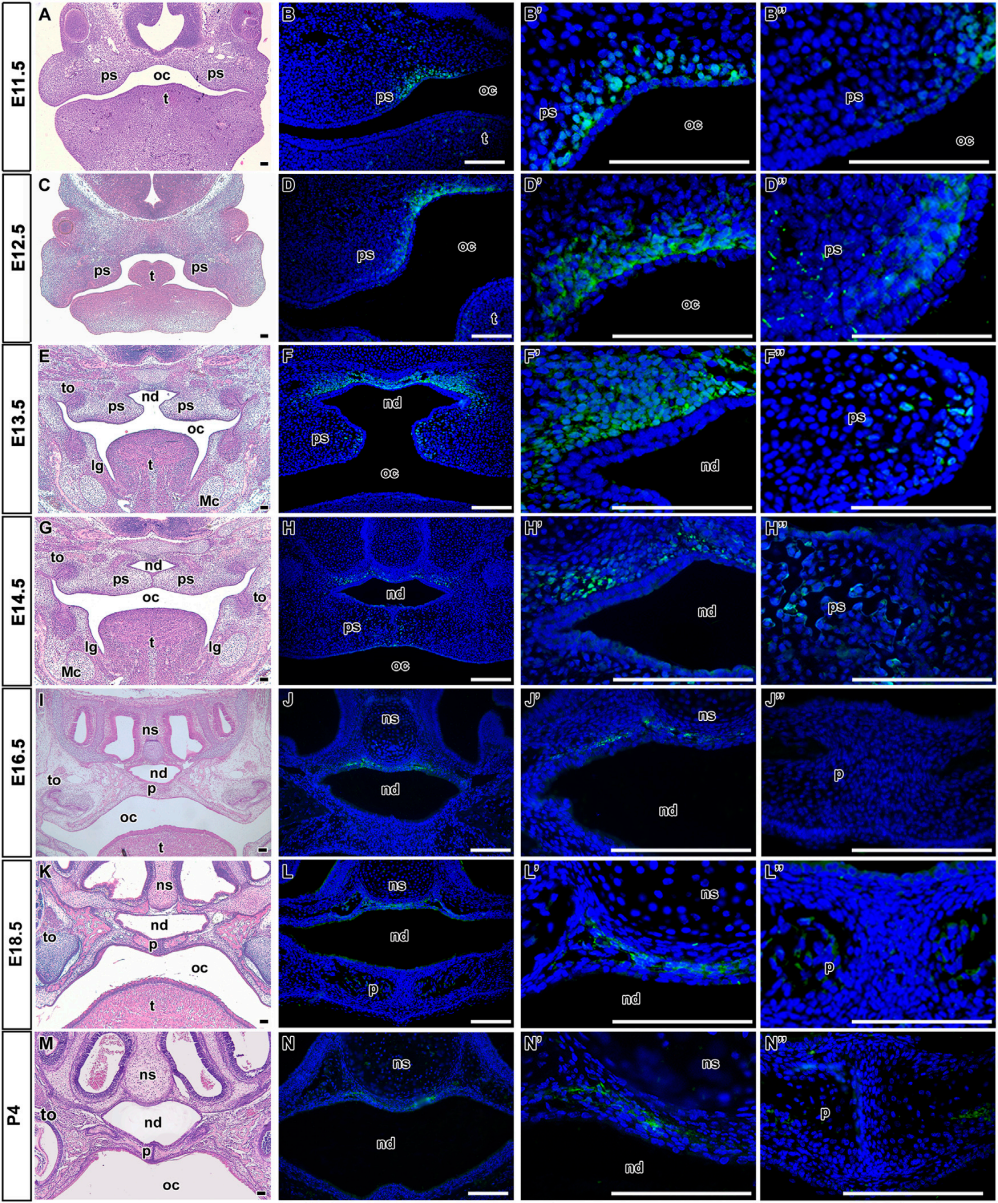
Distribution of LGR5-positive cells during palatogenesis. Hematoxylin-Eosin staining of transversal head sections at prenatal and postnatal stages with focus on the palatal area **(A,C,E,G,I,K,M)**. At E11.5, LGR5-positive cells are located adjacent to the epithelium in the dorsal mesenchyme of forming palatal shelves **(** **B** and **B′,B´´** in detail**)**. Later at E12.5 and E13.5, LGR5-positive signal is present in the dorsal mesenchyme surrounding future nasopharyngeal duct **(** **D,F** and **D´,F´** in detail**)** and in the mesenchyme of developing palatal shelves **(D´´,F´´)**. Next, LGR5-positive cells are situated in the mesenchyme surrounding the nasal septum ventrally from nasal cartilage at E14.5 **(** **H,H´** in detail**)**, E16.5 **(** **J,J´** in detail**)**, E18.5 **(** **L,L´** in detail**)** and P4 **(** **N,N´ in detail)**. Several LGR5-positive cells are located in the ventral palatal epithelium or in the mesenchyme of palatal shelves at E14.5 **(H´´)**. Weak to no expression of LGR5 is found in the epithelium and mesenchyme of palatal shelves at E16.5 **(J´´)**, E18.5 **(L´´)** and P4 **(N´´)**. lg, lingual groove; Mc, Meckel’s cartilage; nd, nasopharyngeal duct; ns, nasal septum; oc, oral cavity; p, palate; ps, palatal shelve; t, tongue; th, tooth. Scale bars = 100 μm.

**FIGURE 2:**
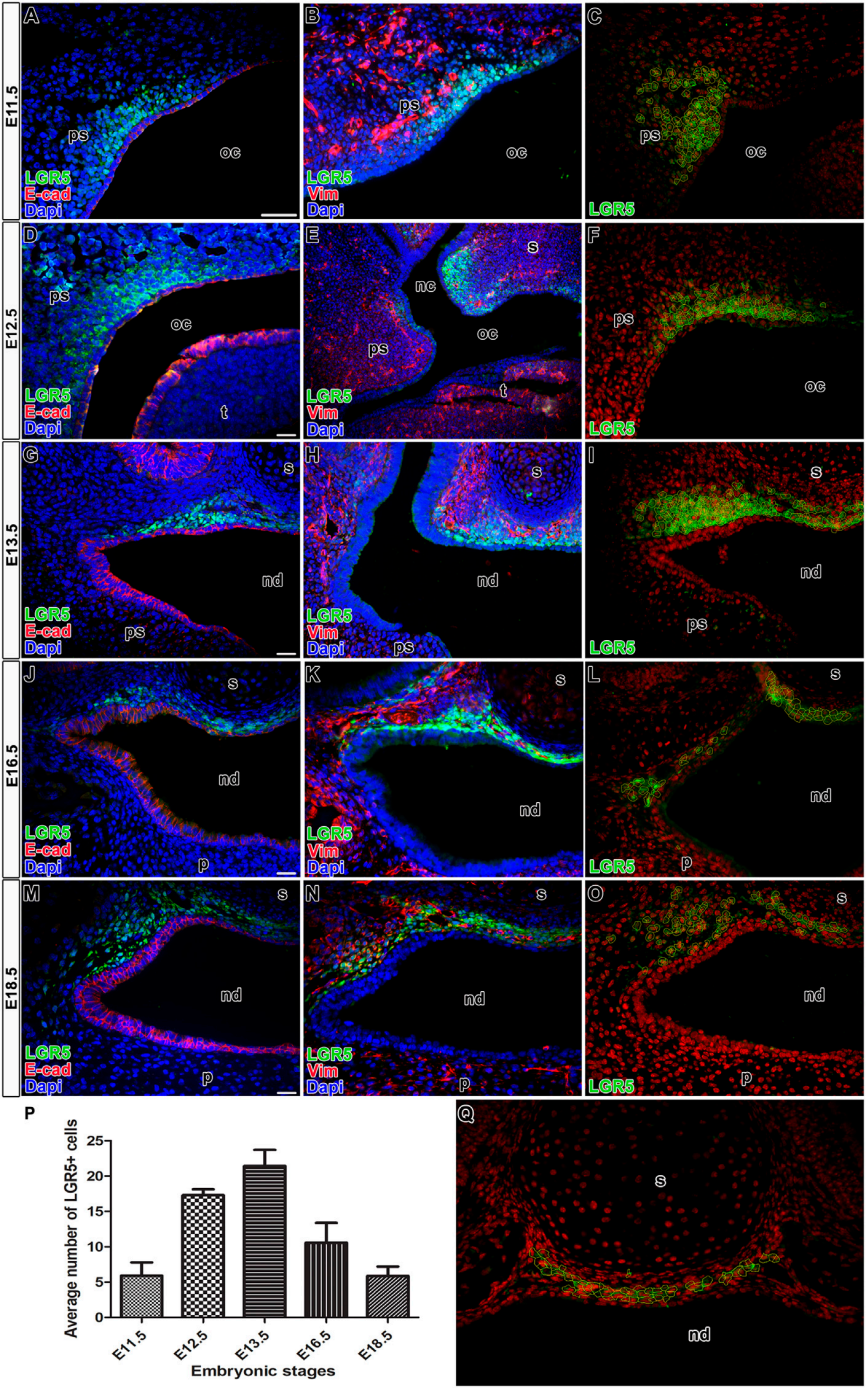
Co-expression of LGR5 and epithelial or mesenchymal marker in palatal areas. Transversal sections of mice heads through the nasopharyngeal duct area double stained by anti-EGFP and anti-E-cad as an epithelial marker **(A,D,G,J,M)** or anti-EGFP and anti-vimentin as a mesenchymal marker **(B,E,H,K,N)**. There is no overlap of expression of LGR5 (anti-EGFP) and epithelial marker E-cad at E11.5 **(A)**, E12.5 **(D)**, E13.5 **(G)**, E16.5 **(J)**, E18.5 **(M)**. Co-expression of LGR5 and mesenchymal marker vimentin is located in the dorsal mesenchyme of forming palatal shelves adjacent to the epithelium at E11.5 **(B)**, in mesenchyme surrounding future nasopharyngeal duct at E12.5 **(E)**, E13.5 **(H)** and in the mesenchyme surrounding the nasal septum at E16.5 **(K)** and E18.5 **(N)**. Examples of analyzed cell populations of LGR5-positive cells in the nasopharyngeal duct area and underlaying nasal septum **(C,F,I,L,O,Q)**. Graph displays quantification of LGR5-positive cells in nasopharyngeal duct area **(P)**. The highest number of positive cells was observed at E13.5. nc, nasal cavity; nd, nasopharyngeal duct; oc, oral cavity; p, palate; ps, palatal shelve; t, tongue; s, septum. Scale bars = 100 μm.

In the palatal area, LGR5-positive cells were found from early prenatal stages (Figure 1). At the time of palatal shelves initiation (E11.5) from the maxillary prominences (Figure 1A), LGR5-positive cells were located in the dorsal mesenchyme of forming palatal shelves adjacent to the epithelium with the most robust expression surrounding the epithelial bending (Figures 1B, B´; Figures 2A, B). In the ventral area of growing palatal shelves, no or weak LGR5 expression was seen (Figure 1B´´). During the next stages (E12.5 and E13.5) of craniofacial development, the palatal shelves grew vertically along the tongue (Figures 1C, E), and the expression of LGR5 was found in the dorsal mesenchyme surrounding the future nasopharyngeal duct (Figures 1D, D´, F, F´; Figures 2D, E, G, H) and in the mesenchyme of developing palatal shelves, especially in their medial tips (Figures 1D´´, F´´). At the stage when the palatal shelves underwent horizontalisation followed by their fusion (E14.5; Figure 1G), LGR5-positive cells were situated in the mesenchyme ventrally from nasal septum and also in the mesenchyme located dorsally from the nasopharyngeal duct (Figures 1H, H´). Several LGR5-positive cells were also detected in the palatal epithelium and the mesenchyme of palatal shelves close palatal seam (Figures 1H, H´´). Later in development (E16.5, E18.5, and P4) (Figures 1I, K, M; Figures 2J, K, M, N), the expression of LGR5 was mainly found in the mesenchyme located ventral to the nasal septum. The signal was weaker in the palatal area in comparison to early developmental stages, indicating its possible association with palatal morphogenesis (Figures 1J, J´, L, L´, N, N´) and palatal shelve shaping rather than mesenchymal cells maintenance (Figures 1J, J´´, L, L´´, N, N´´). Quantification of LGR5-positive cells was carried out at E11.5 (Figure 2C), E12.5 (Figure 2F), E13.5 (Figure 2I), E16.5 (Figure 2L), and E18.5 (Figure 2O), and it revealed their highest number in the nasopharyngeal area at early stages, especially at E12.5 and E13.5 (Figure 2P).

### LGR5-positive cells are surrounding the vomeronasal organ

**FIGURE 3:**
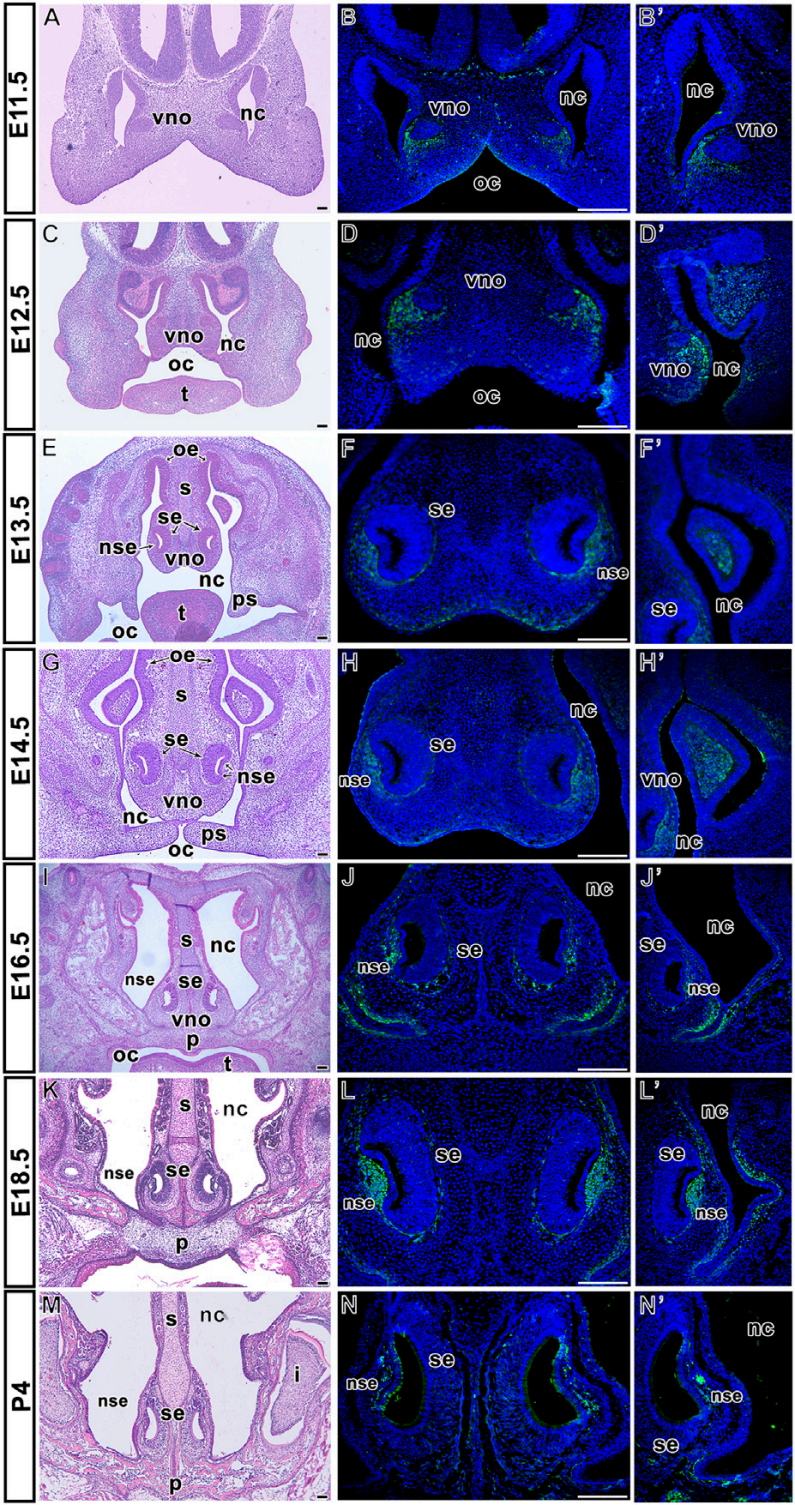
Distribution of LGR5-positive cells in the vomeronasal organ area. Hematoxylin-Eosin stained sections of vomeronasal organ at several prenatal and one postnatal embryonic stage **(A,C,E,G,I,K, M)**. Expression of LGR5 is located in the mesenchyme underlying the lateral epithelium of the nasal cavity at E11.5 **(** **B,B´** in detail**)** and E12.5 **(D)**. LGR5-positive cells are observed in the mesenchyme of developing nasal conchae at E12.5 **(D´)**, E13.5 **(F´)** and E14.5 **(H´)**. Signal of LGR5-positive cells is also situated in the mesenchyme of both sensory and non-sensory epithelium of VNO at E13.5 **(F)** and E14.5 **(H)**. At the next stages, LGR5-positive cells are located in the mesenchyme of non-sensory epithelium of VNO at E16.5 **(J)**, E18.5 **(L)** or P4 **(N)**, as well as in the lateral mesenchyme surrounding the nasal cavity, and in the mesenchyme surrounding ventral epithelium of the nasal cavity at E16.5 **(J´)**, E18.5 **(L´)** and P4 **(N´)**. i, incisor; nc, nasal cavity; ns, nasal septum; nse, non-sensory epithelium; oe, oral epithelium; oc, oral cavity; p, palate; ps, palatal shelves; se, sensory epithelium; t, tongue; vno, vomeronasal organ. Scale bars = 100 μm.

The vomeronasal organ (VNO) begins to develop around E11. At early embryonic stages (E11.5, E12.5) (Figures 3A, C; Supplementary Figures S2A–D´), we observed LGR5 expression in the mesenchyme underlying the ventral epithelium of the nasal septum (Figure 3B, D; Supplementary Figures S2A–D´), in the mesenchyme around respiratory epithelium of developing VNO, and in the surrounding developing nasal conchae (Figures 3B, B´, D, D´; Supplementary Figures S2A´–D´). At E13.5 (Figure 3E; Supplementary Figures S2E–F´), LGR5-positive cells were found along both the sensory and non-sensory epithelium of VNO (Figure 3F; Supplementary Figures S2E, F), as well as in the ventral mesenchyme of the nasal septum and the area of the branching nasal cavity (Figures 3F, F´; Supplementary Figures S2E´, F´). At E14.5 (Figure 3G), LGR5 was present in the same areas as at earlier developmental stages. It was also found in the mesenchyme localised on both sides of the ventral part of the nasal cavity, along the sides of the nasal septum, and in the mesenchyme of the conchae (Figures 3H, H´). At E16.5 and E18.5 (Figures 3I, K; Supplementary Figures S2G–J´), LGR5 expression was maintained in the mesenchyme of the non-sensory epithelium of VNO (Figures 3J, L; Supplementary Figures S2G, H, I, J) and the mesenchyme surrounding the nasal cavity. In the ventral part of the nasal septum, LGR5-positive cells were located in the lateral mesenchyme (Figures 3J´, L´; Supplementary Figures S2G´, H´, I´, J´). In contrast, a low number of LGR5-positive cells were observed around the VNO at early postnatal day 4 (P4) (Figures 3M, N, N´).

### LGR5-positive cells are asymmetrically distributed in the mesenchyme surrounding the lingual groove

**FIGURE 4:**
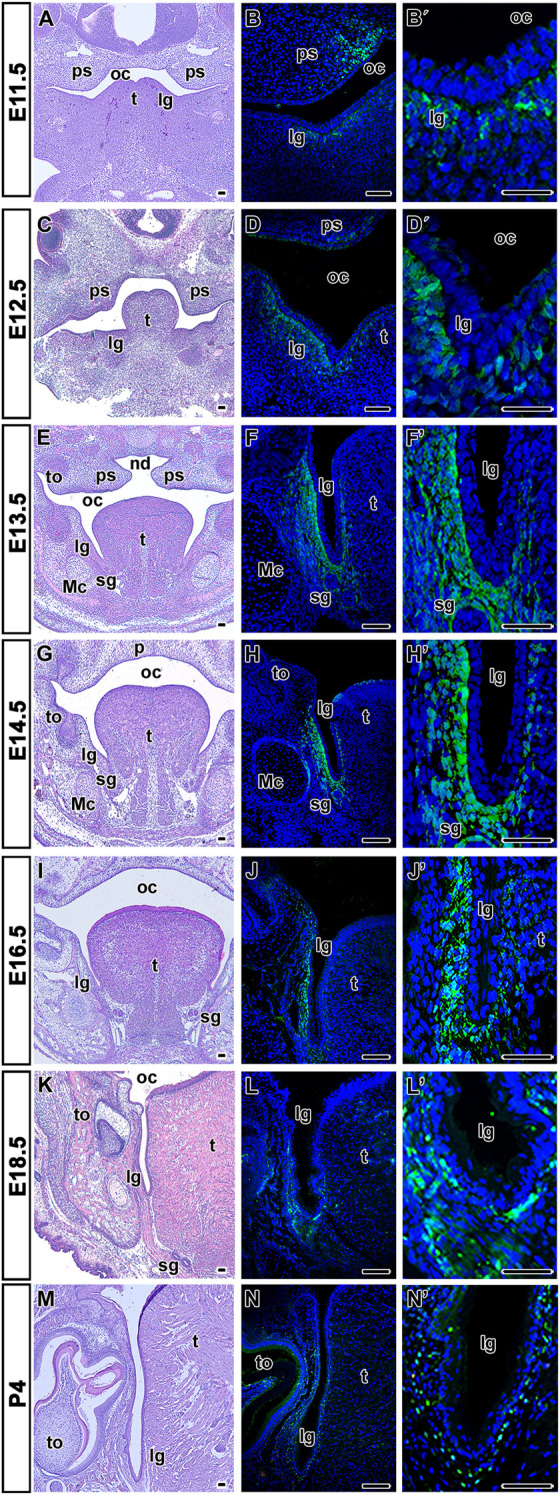
Localization of LGR5-positive cells during lingual groove development. Transversal sections of heads through the lingual groove area stained by Hematoxylin-Eosin at several prenatal and one postnatal stage **(A,C,E,G,I,K,M)**. LGR5-positive cells are situated in the mesenchyme of labial and lingual side of developing groove at E11.5 **(** **B,B´** in detail**)** and E12.5 **(** **D,D´** in detail**)**. Population of LGR5-positive cells is situated in the labial and lingual side of groove mesenchyme and also around epithelium of salivary glands at E13.5 **(** **F,F´** in detail**)**, E14.5 **(** **H,H´** in detail**)**, E16.5 **(** **J, J´** in detail**)**, E18.5 **(** **L,L´** in detail**)** and P4 **(** **N,N´** in detail**)**. lg, lingual groove; Mc, Meckel’s cartilage; oc, oral cavity; ps, palatal shelve; sg, salivary gland; t, tongue; th, tooth. Scale bars = 100 μm.

The tongue is formed from two rostral prominences in the primitive stomodeum and a pharyngeal prominence in the adjacent caudal area—their fusion results in the formation of a single protrusion surrounded by the lingual groove. The lingual groove is a structure that starts to develop along the tongue at E11.5 (Figure 4A). The expression of LGR5 was observed in the mesenchyme surrounding the groove area from this early stage (Figures 4B, B´). However, starting at embryonic stage E12.5 (Figure 4C), the LGR5-positive cells were asymmetrically distributed with more positive cells located in the labial mesenchyme of the developing lingual groove (Figures 4D, D´). Several LGR5-positive cells were also localised on the lingual side of the groove (Figure 4D´). Interestingly, LGR5 expression increased with lingual groove extension. The same pattern of LGR5 expression was detected in the lingual groove area at later embryonic stages (E13.5-E16.5) (Figures 4E, G, I), with lower expression observed on the lingual side and a stronger signal preserved in the mesenchyme of the labial side of the lingual groove and around the epithelium of the salivary glands. This LGR5^+^ cell population extended to the area close to Meckel’s cartilage (Figures 4F, F´, H, H´, J, J´). At E18.5 (Figure 4K) and P4 (Figure 4M), LGR5 expression decreased in the labial mesenchyme of the lingual groove; however, a few LGR5-positive cells could still be detected around groove epithelium (Figures 4L, L´, N, N´).

### Lineage tracing of LGR5-positive cells in the upper jaw

**FIGURE 5:**
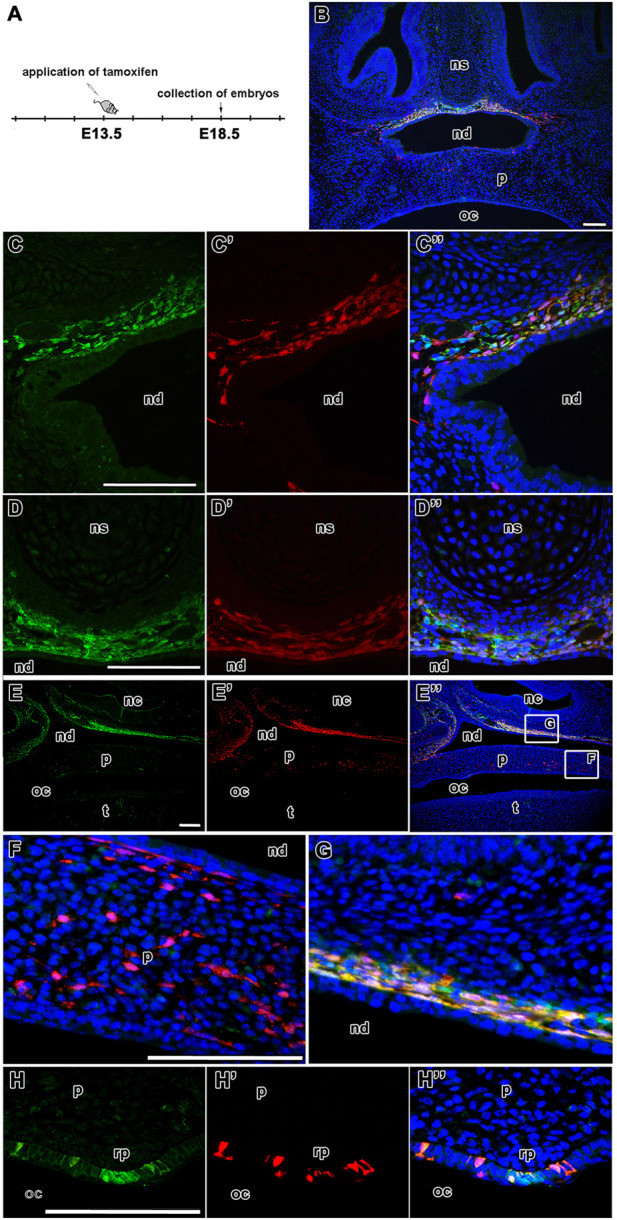
Fate of LGR5-positive cells during palatogenesis. Timeline of tamoxifen application and collection of samples **(A)**. Transversal sections of lineage tracing in the developing nasopharyngeal duct and palate of *Lgr5-EGFP-CreERT2* (green color) and *Rosa26-tdTomato* reporter mice (red color) at E18.5 after tamoxifen administration **(B)**. The expression of LGR5/EGFP positive cells at final time point at E18.5 in the mesenchyme beyond epithelium of nasopharyngeal duct **(C, D)**. LGR5/tdTomato positive cells at time point from E13.5 to E18.5 **(C´,D´)** and the overlap of LGR5/EGFP positive cells and LGR5/tdTomato positive cells at E18.5 **(C´´,D´´)**. Sagittal section of lineage tracing in the caudal region of palate **(E, E´,E´´)**. Higher-magnification of palatal area with LGR5/tdTomato positive cells in mesenchyme of palate **(F)** and an overlap of LGR5/EGFP and LGR5/tdTomato cells in the mesenchyme of nasopharyngeal duct **(G)**. Surprisingly, LGR5/EGFP positive cells are located in the epithelium of *rugae palatinae* **(H´)**. Moreover, several LGR/tdTomato cells are situated in the similar area of rugae palatinae, thus there is an overlap of LGR5/EGFP and LGR5/tdTomato cells **(H´´)**. nc, nasal cavity; nd, nasopharyngeal duct; ns, nasal septum; oc, oral cavity; p, palate; rp, rugae palatinae; t, tongue. Scale bars = 100 μm.

**FIGURE 6:**
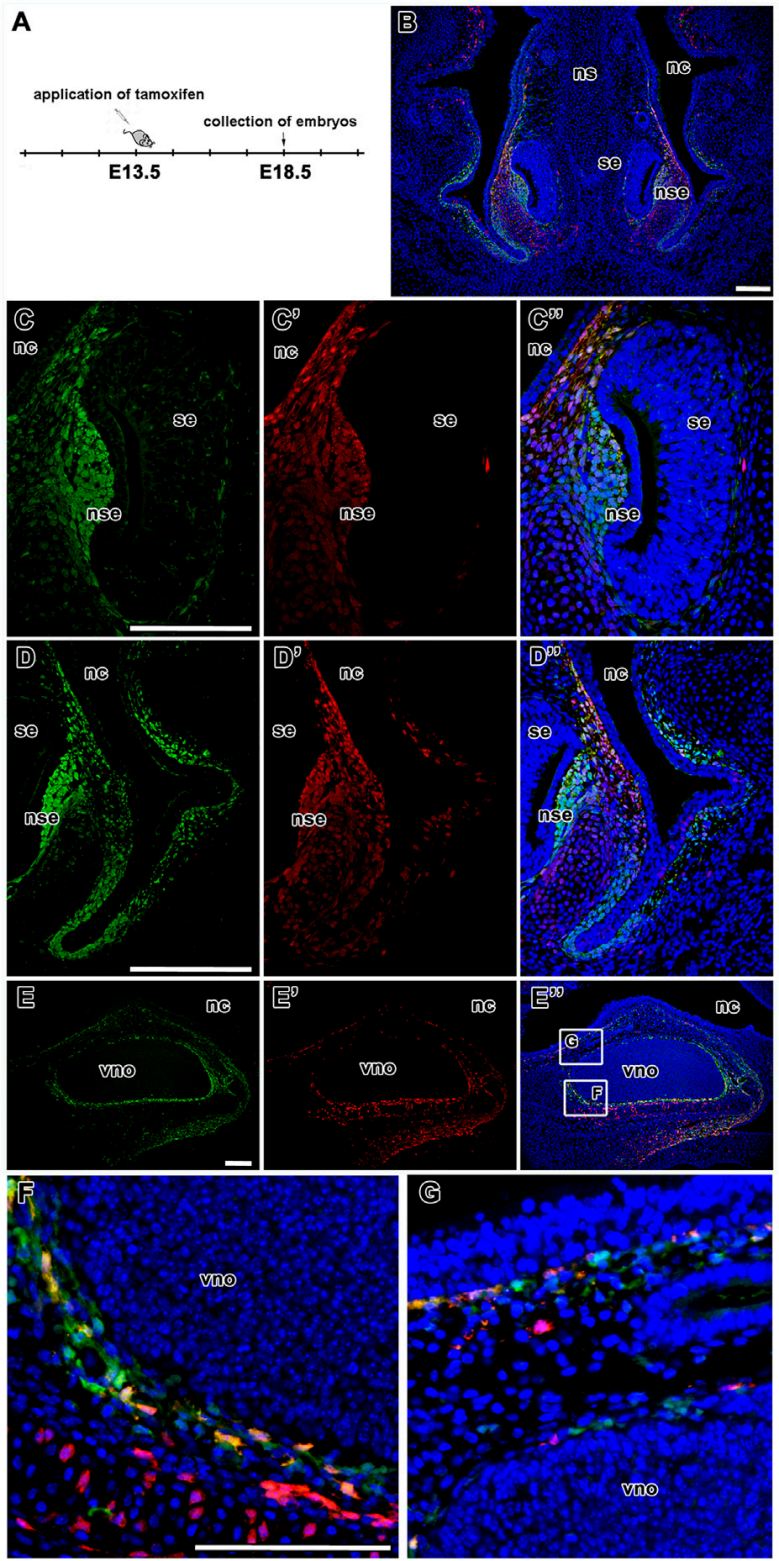
Lineage tracing of LGR5-positive cells during development of vomeronasal organ. Timeline of tamoxifen application and collection of embryos **(A)**. Low-magnification image of VNO after tamoxifen induction and embryos collection at E18.5. LGR5/EGFP positive cells are represented by green color, LGR5/tdTomato positive cells are represented by red color and the overlap of LGR5/EGFP positive cells and LGR5/tdTomato positive cells is represented yellow color **(B)**. Higher-magnification of lineage tracing in the VNO area **(C-D´´)**. The LGR5/EGFP positive cells are situated in the mesenchyme underlying the lateral non-sensory epithelium, several cells are localized also around sensory epithelium of VNO **(C,C´´)** and in the mesenchyme underlying lateral epithelium nasal cavity. They are also spread through the mesenchyme in the ventral part of nasal septum **(D,D´´)**. Similar distribution like LGR5/EGFP positive cells is observed in LGR5/tdTomato positive cells. These cells are situated in mesenchyme of non-sensory epithelium **(C´,C´´)**, in the mesenchyme underlying lateral epithelium nasal cavity **(D´,D´´)** where is their overlap detected **(C´´,D´´)**. In contrast with LGR5/EFP positive cells, the LGR5/tdTomato cells are not observed in the mesenchyme of sensory epithelium of VNO **(C´,C´´)**. These observations are confirmed also by sagittal sections of vomeronasal organ in low-magnification pictures **(E, E´, E´´** and in detail **F,G)**. nc, nasal cavity; nd, nasopharyngeal duct; ns, septum; nse, non-sensory epithelium; oc, oral cavity; se, sensory epithelium; vno, vomeronasal organ. Scale bars = 100 μm.

**FIGURE 7:**
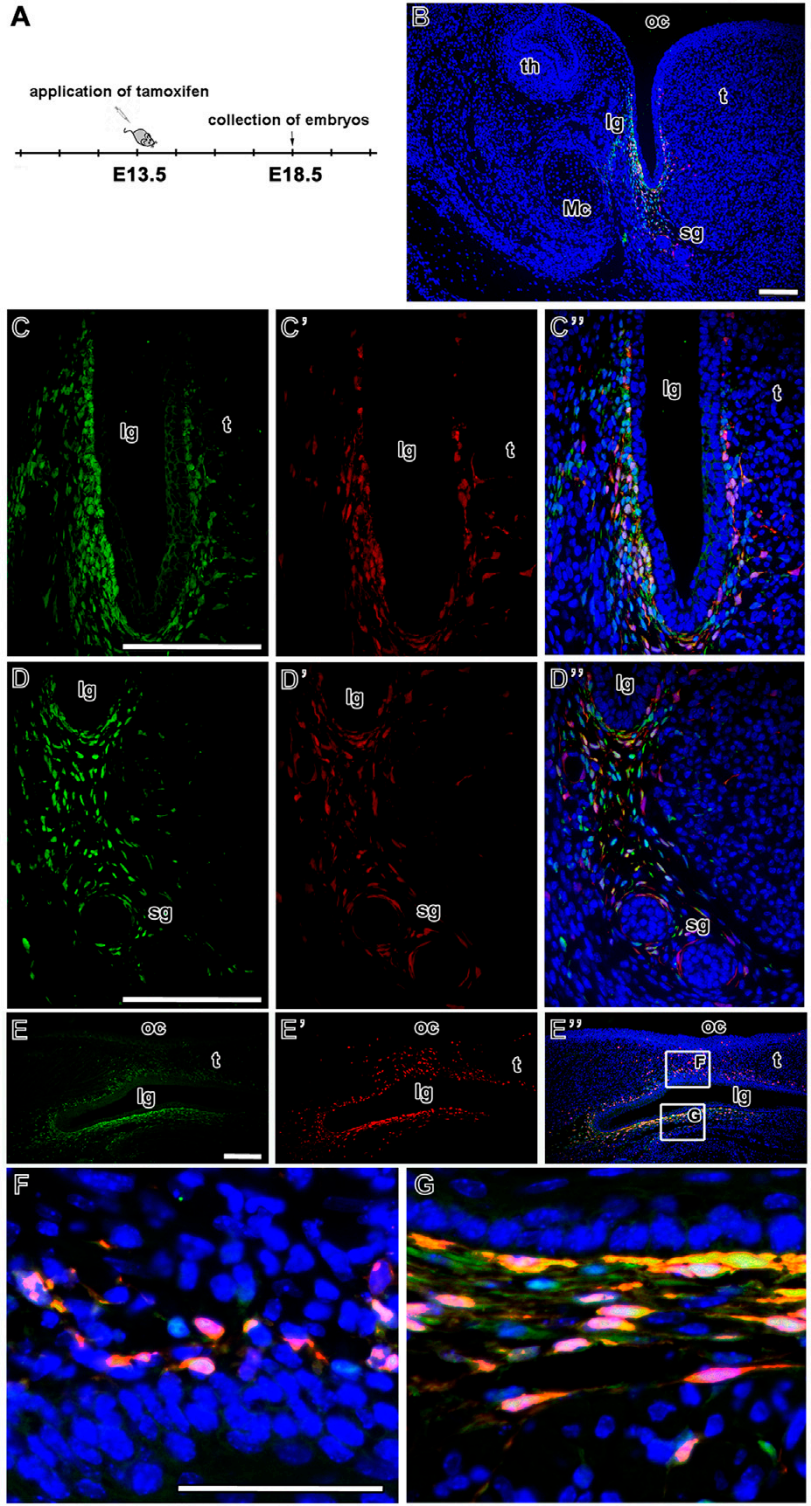
Fate of LGR5-positive cells during development of lingual groove. Timeline of tamoxifen induction and collection of embryos **(A)**. Low-magnification image of the area of lingual groove after lineage tracing analysis. The overlap of LGR5/EGFP positive cells (green color) and LGR5/tdTomato positive cells (red color) represented by yellow color **(B)**. Higher-magnification of fate mapping of LGR5-positive cells **(C-D´´)**. The LGR5/EGFP positive cells are situated in the mesenchyme of labial side of the lingual groove, some of them are localized into the lingual side of groove **(C,C´´)** or they are spread up to the outlets of salivary glands **(D,D´´)**. The LGR5/tdTomato positive cells are distributed in the similar way, in the labial and lingual mesenchyme of the groove **(C´,C´´)** and in the mesenchyme around salivary glands **(D´,D´´)**. There is observed an overlap of LGR5/EGFP positive cells and LGR5/tdTomato positive cells in above mentioned regions **(C´´,D´´)**. The sagittal sections of lingual groove confirm distribution pattern of LGR5-positive cell in the area of lingual groove **(E,E´, E´´** and in detail **F, G)**. lg, lingual groove; Mc, Meckel´s cartilage; oc, oral cavity; sg, salivary gland; t, tongue; th, tooth. Scale bars = 100 μm.

Next, we followed the fate of LGR5-expressing cells in the upper and lower jaw while using *Lgr5-EGFP-IRES-CreERT2* animals crossed to *Rosa26-tdTomato* reporter mice. Several time frames were used for LGR-positive cells labelling and tracing: (1) tamoxifen injection at E13.5, sample analysis at E18.5 (Figures 5–7); (2) tamoxifen injection at E13.5, sample analysis at E15.5 or E16.5 (Supplementary Figures S3–S5 and data not shown); and (3) tamoxifen injection at E12.5 with sample analysis at E18.5 (Supplementary Figure S6). The shortest time point was also used for whole-mount native fluorescence visualisation in the maxillary regions (Figure 9; Supplementary Figure S3E). As expression of LGR5 was the strongest at E13.5 (Figures 1, 3), we administered tamoxifen at this stage and followed the fate of the LGR5-positive cell population at later time points. We also compared these results with labelling at E12.5 and subsequent analysis at E18.5 (Supplementary Figure S6). Unfortunately, we were not able to obtain postnatal stages for lineage tracing as tamoxifen treatment during pregnancy interfered with newborn deliveries.

**FIGURE 8:**
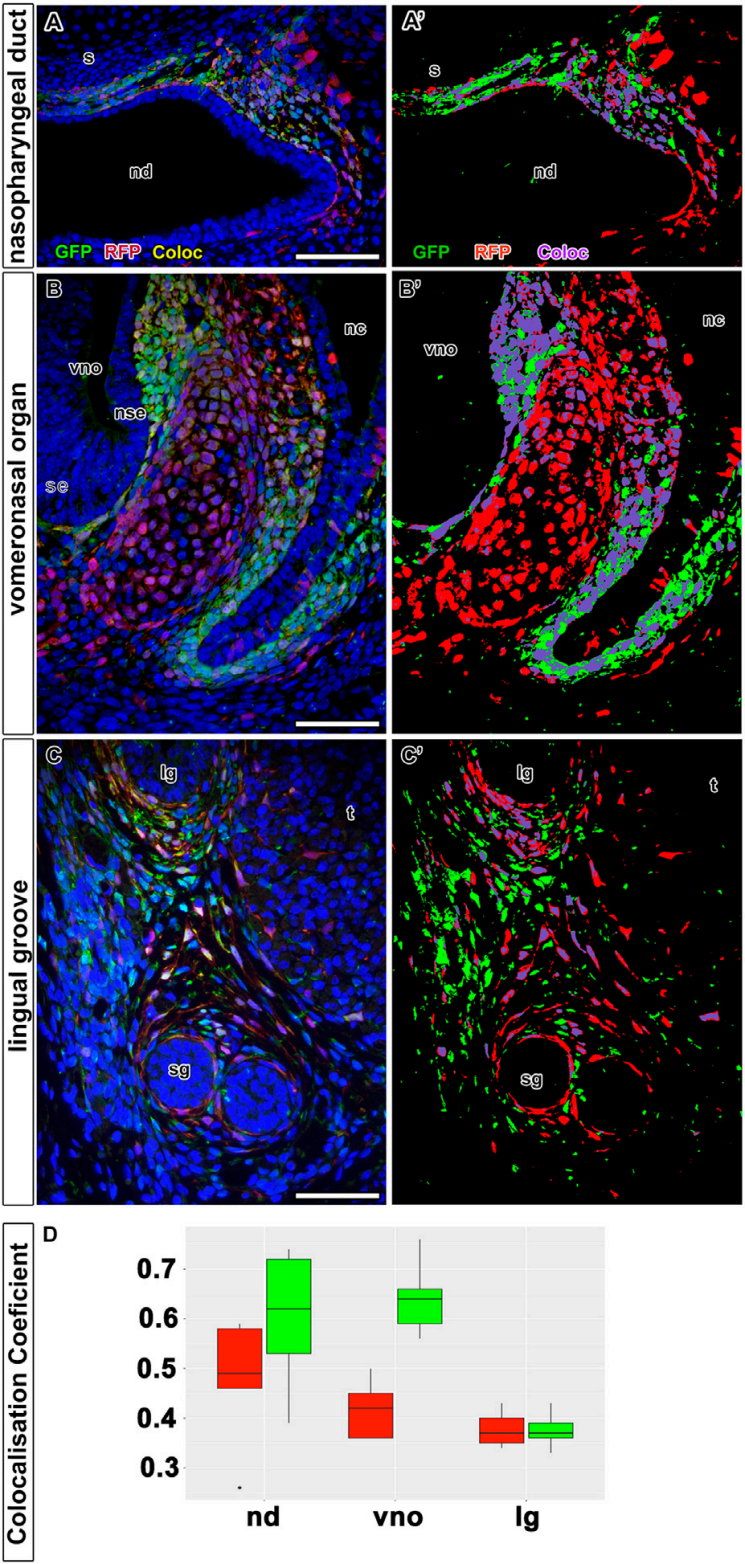
Colocalization analysis of LGR5/EGFP and LGR5/tdTomato positive cells on tissue sections. Quantitative analyses of colocalization between LGR5/EGFP positive cells (green color) and LGR5/tdTomato positive cells (red color) on immunoassayed tissue sections. Nuclei counterstained with DAPI are in blue. In left panel, there are representative images displaying overlayed channels red, green and blue **(A,B,C)**. Images in right panel **(A´,B´,C´)** are corresponding to binary masks images from the left panel demonstrating classified pixels above the threshold, which were used for colocalization coefficient calculation. Pixels for LGR5/EGFP channel are in green, LGR/tdTomato channel in red and co-localized pixels are labeled with turquoise color. **(D)** Boxplots summarizing colocalization analysis from all images of each group: nd, nasopharyngeal duct; vno, vomeronasal organ; lg, lingual groove. Boxplots filled with red color representing colocalization coefficient for red channel, and boxplots filled with green color representing colocalization coefficient for green channel. nd displays the highest level of colocalization for both channels. On the other side, sample lingual groove exhibit the lowest level of colocalization in both channels. vno exhibit the higher level of colocalization in green channel, whereas red channel displays lower level of colocalized pixels. lg, lingual groove; nd, nasopharyngeal duct; nc, nasal cavity; nse, non-sensory epithelium; s, septum; se, sensory epithelium; sg, salivary glands; t, tongue; vno, vomeronasal organ.

Using the first time protocol (analysis at E18.5) (Figures 5A, B), cells expressing LGR5, i.e. EGFP-positive cells, were located in the mesenchyme surrounding the nasal septum ventrally from nasal septum and also more laterally in the mesenchymal cells underlying the dorsal epithelium of the nasopharyngeal duct (Figures 5C, D). Expression of tdTomato was found in the same areas (Figures 5C´, D´). We observed the highest overlap of tdTomato (red) and EGFP (green) fluorescence in the nasopharyngeal duct area (Figures 5B, C´´, D´´ and Figures 8A, A´); the colocalisation was detected along the entire length of the developing mesenchyme surrounding the nasopharyngeal duct (Figures 8A, A´). This was quantified by the colocalisation coefficient. Sections from the nasopharyngeal duct revealed the highest level of colocalisation among the craniofacial structures quantified in both, i.e. red and green, fluorescent channels (boxplots in Figure 8D).

The distribution of labelled cells was further analysed on sagittal sections (Figures 5E-H´´) where scattered tdTomato-positive cells were observed in the palatal shelves (Figures 5E´, F). In contrast, double labelled cells were located more dorsally in the mesenchyme surrounding the nasopharyngeal duct (Figure 5G). Moreover, EGFP-positive cells were found in the area of *rugae palatinae* with significant overlap with tdTomato fluorescence in the rugal epithelium (Figures 5H, H´´).

A similar pattern of distribution of tdTomato^+^ and EGFP^+^ cells was observed in the palatal area at an earlier collected time point (from E13.5 to E15.5) (Supplementary Figure S3A). Numerous cells at E15.5 expressing LGR5, i.e. EGFP-positive cells, co-expressed tdTomato (Supplementary Figures S3B-D´´), suggesting persistence of LGR5-positive cells in the analysed area. This finding was confirmed by 3D images of the craniofacial area (Supplementary Figures S3E, E´, E´´; Supplementary Video S1).

**FIGURE 9:**
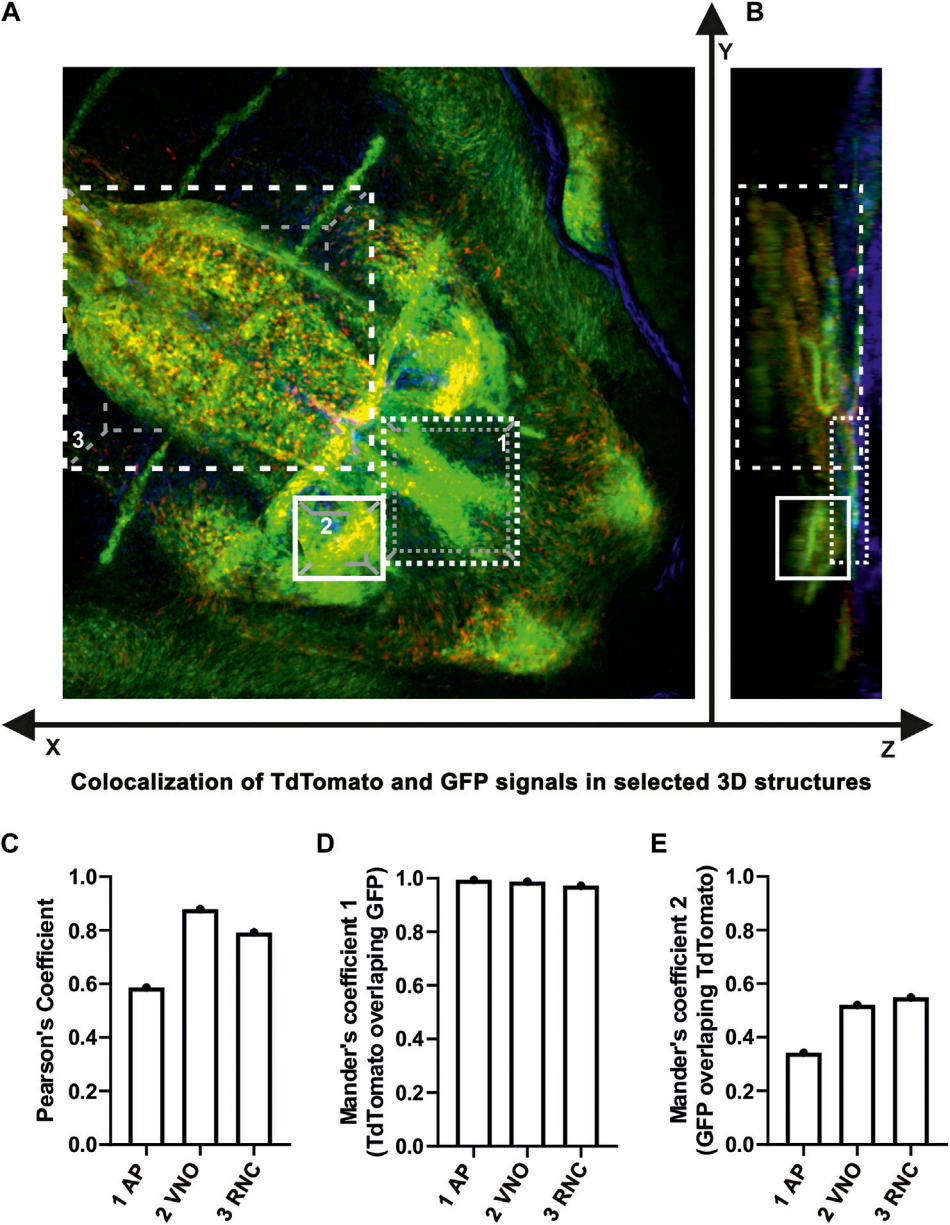
**(A,B)** Colocalization analysis of LGR5/EGFP and LGR5/tdTomato positive cells in 3D. Quantitative measurements of colocalization between LGR5/EGFP positive cells (green color) and LGR5/tdTomato positive cells (red color) in three distinct morphological structures of the upper jaw region. Nuclei counterstained with DAPI are in blue. In the upper part of the panel, analyzed structures are graphically dissected in three dimensions of the Z-stack image: 1) anterior palate (AP), 2) area of vomeronasal organ (VNO), 3) roof of nasopharyngeal cavity (RNC). **(C,D,E)** In the lower part of the panel, there are bar charts displaying measured tdTomato/EGFP overlap in individual structures in Mander’s coefficient and both variations of Pearson’s coefficients. For the sake of figure compactness, analyzed structures are designated in short within the bar charts.

In addition, 3D analysis of the overlap of fluorescence signals in a whole-mount upper jaw at E15.5 (tamoxifen administration at E13.5) exhibited strong colocalization of tdTomato- and EGFP-positive cells in agreement with our previous results (Figures 9A–E). The majority of cells that underwent Cre-mediated recombination of the transcriptional stop box maintained LGR5/EGFP expression at the time of sampling, indicating persistence of LGR5-positive cells within the timeframe (Mander’s colocalisation coefficient 1, Figure 9D). Interestingly, there was a clear difference in the ratio of double-positive cells among selected morphological areas. The anterior region of the palate (AP) displayed less overlap of tdTomato and EGFP signals when compared to the vomeronasal region (VNO) and roof of the nasopharyngeal cavity (RNC) (Mander’s colocalisation coefficient 2, Figure 9E), indicating a different abundance of LGR5-positive cells in individual parts of the upper jaw at the time of tamoxifen induction and possible later onset of LGR5 expression in the anterior palate. Next, we analysed the fate of LGR5-positive cells in the area of VNO (Figures 6A, B). EGFP^+^ cells were found in the underlying mesenchyme lateral to the non-sensory epithelium, and a few EGFP-positive cells were also detected around the sensory epithelium (Figure 6C). In contrast, tdTomato-positive cells were visible in the mesenchyme surrounding VNO non-sensory epithelium (Figure 6C´). Furthermore, EGFP-positive cells were also found in the mesenchyme underlying the lateral epithelium of the nasal cavity, and they spread through the mesenchyme to the ventral part of the nasal septum (Figure 6D). On the other hand, tdTomato-positive cells were found in the mesenchyme adjacent to the epithelium lining the nasal cavity (Figure 6D´). Consequently, the overlap of fluorescent signals was observed in the mesenchyme underlying the non-sensory epithelium of VNO and in the mesenchyme underlying the ventral epithelium of the nasal cavity attached to the *regio respiratoria* (Figures 6B, C´´, D´´ and Figures 8B, B´). VNO area displayed higher levels of colocalisation of “green” cells (cells actively transcribing the *Lgr5* locus) with red fluorescence when compared to colocalisation of tdTomato expressing (red) cells with green fluorescence (boxplot in Figure 8D). This might indicate the transition of originally LGR5-positive to LGR5-negative cells. This is supported by the fact that images from this part of the embryo have the highest proportion of pixels in the red channel, which indicates that there were a large number of cells that expressed LGR5 in the past but no longer express it (Figure 8D). Nevertheless, EGFP/tdTomato double-positive cells were observed in the mesenchyme of the lateral nasal cavity (Figures 6B, D´´; Figures 8B, B´). Sagittal sections revealed a close proximity of EGFP-positive cells to the VNO epithelium. In contrast, the tdTomato signal was farther from the epithelium, and the red cells were dispersed to the dorsal mesenchyme (Figures 6E–G). It indicates the migration of numerous cells that lost LGR5 expression from the area adjacent to the VNO epithelium during VNO development. However, some cells remined in their original location and retained LGR5 expression, i.e., EGFP positivity.

At shorter time point (from E13.5 to E15.5) (Supplementary Figure S4A), LGR5/tdTomato positive cells were mainly observed around the non-sensory epithelium of VNO. Several cells were also found around the sensory epithelium (Supplementary Figures S4B, C´) and in the mesenchyme of the underlying lateral epithelium of the nasal cavity (Supplementary Figures S4B, D´). LGR5/EGFP positive cells located in the area of developing vomeronasal organ (Supplementary Figures S4B, C, D) significantly overlapped with LGR5/tdTomato positive cells in the mesenchyme of non-sensory epithelium of VNO (Supplementary Figures S4B, C´´) and also in the mesenchyme of the underlying lateral epithelium of the nasal cavity (Supplementary Figure S4B, D´´). More LGR5/tdTomato cells were found around the sensory epithelium of VNO than at later time points (collection at E18.5). This suggested that the cells induced at E13.5 remained at the same place in the non-sensory area while only some cell dispersion could be detected at E15.5.

At the last time point (from E12.5 to E18.5) (Supplementary Figure S6A), LGR5/tdTomato positive cells were observed in the mesenchyme surrounding the nasopharyngeal duct (Supplementary Figures S6B´, B´´). LGR5/EGFP positive cells were localised in the same areas (Supplementary Figures S6B´, B´´). There was a significant overlap of LGR5/tdTomato and LGR5/EGFP positive cells in all analysed upper jaw areas (Supplementary Figures S6B´´). In the VNO area, LGR5/EGFP-positive cells were situated in the mesenchyme around the non-sensory epithelium of VNO and the mesenchyme underlying the lateral epithelium of the nasal cavity (Supplementary Figures S6CC´´, DD´´). The LGR5/tdTomato-positive cells were observed in the same areas, but they were more dispersed around both the non-sensory and sensory epithelium (Supplementary Figures S6C´, C´´, D´, D´´). There was also a significant overlap of LGR5/EGFP and LGR5/TtdTomato-positive cells in the mesenchyme of the lateral nasal cavity and around the non-sensory epithelium of VNO (Figures 6C´´, D´´).

### Lineage tracing of LGR5-positive cells in the tongue groove area

Finally, we analysed the distribution of EGFP- and tdTomato-positive cells (and their possible colocalisation) in the area of the developing tongue. In the lingual groove mesenchyme, there was an overlap of LGR5/EGFP-positive cells and LGR5/tdTomato cells when tamoxifen was injected at E13.5 and embryos collected at E18.5 (Figure 7A). Overlap of both signals was mainly observed in the more lateral area of the mesenchyme (Figure 7B). The majority of LGR5/EGFP-positive cells were located in the mesenchyme adjacent to the epithelium in the tip of the lingual groove. However, some of these cells were also visible more ventrally (Figures 7C, C´´, D, D´´). Cells expressing LGR5/tdTomato expanded into the lingual (medial) mesenchyme of the groove inside the tongue (Figures 7C´, C´´, D´, D´´). In contrast, the tip of the lingual groove was only surrounded by a few LGR5/tdTomato-positive cells. Several red cells were spread more deeply in the lower jaw mesenchyme toward the salivary glands (Figures 7D´, D´´; Figures 8C, C´). The sagittal section of the developing lingual groove revealed dispersion of tdTomato-positive cells to the tongue cells only expressing LGR5/EGFP, i.e. green cells. These cells were located in ventral areas close to the lingual groove epithelium (Figures 7E–G). Our pixel-based signal quantification revealed a lower level of LGR5/EGFP and LGR5/tdTomato colocalisation in the samples from the lingual groove sections (Figure 8D). This indicates that cells in this region are either positive for EGFP or tdTomato, rather than for both markers.

At a shorter time point with Cre activity induction at E13.5 and collection at E16.5 (Supplementary Figure S5A), LGR5/tdTomato-positive cells were found in the labial mesenchyme of the lingual groove. A few cells were also situated on the lingual side of the lingual groove and in the mesenchyme of the tongue (Supplementary Figures S5B, C´, D´). These cells were dispersed up to the salivary gland ducts (Supplementary Figures S5B, D´). The clearest overlap of the EGFP and tdTomato fluorescent signal was found in the labial mesenchyme of the lingual groove (Supplementary Figures S5B, C´´, D´´ compared to C, D). The staining indicates that in the analysed area, LGR5-positive cells persisted and retained their LGR5 expression.

At the last time point (from E12.5 to E18.5) (Supplementary Figure S6), the LGR5/EGFP-positive cells were localised in the mesenchyme of the labial side of the lingual groove, and several cells were also situated in the tongue (Supplementary Figures S6E, E´´, F, F´´). The LGR5/tdTomato-positive cells were also detected in the mesenchyme of the labial side of the lingual groove. There were more LGR5/tdTomato positive cells found in the caudal area of the tongue (Supplementary Figures S6E, E´´, F, F´´). The overlap of LGR5/EGFP- and LGR5/tdTomato-positive cells was detected in the mesenchyme of the labial side of the lingual groove (Supplementary Figures S6E´, F´´).

### Loss of function of *Lgr5* led to a distinct craniofacial phenotype and disruption of tissue fold formation

**FIGURE 10:**
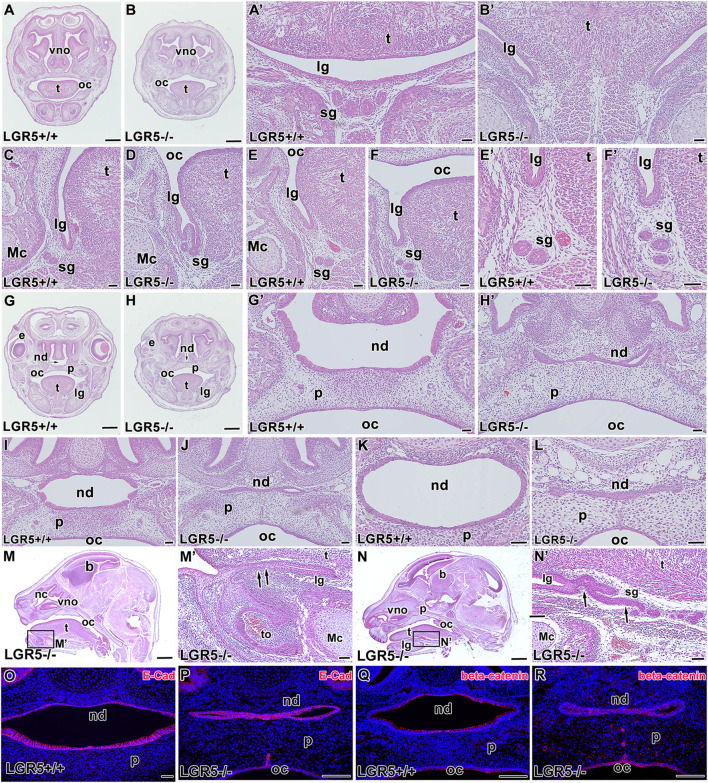
Craniofacial abnormalities of *Lgr5* ^*−/−*^ mice at E16.5. *Lgr5*-deficient mice exhibit several distinct craniofacial defects **(A-L)** as shown on transversal histological sections of *Lgr5*-deficient mice stained by Hematoxylin-Eosin. Low magnification of transversal section of rostral area in *Lgr5* ^*+/+*^ **(A)** and *Lgr5* ^*−/−*^ **(B)** mice. High magnification of tongue area in WT embryo **(A´)** and *Lgr5*-deficient embryo with distinct ankyloglossia **(B´)**. No craniofacial defects can be observed in the area of lingual groove at WT embryo **(C,E,E´)**. Branching of epithelium **(D)** and multiplication of outlets of salivary glands **(F,F´)** was observed in *Lgr5*-deficient mice. Low-magnification image of palate area of *Lgr5* ^*+/+*^ **(G)** and *Lgr5* ^*−/−*^ **(H)** mice. High magnification of developing palate with correctly formed nasopharyngeal duct **(G´)**. Abnormal morphology of nasopharyngeal duct in mutant embryo **(H´)**. Histological section of middle area of nasopharyngeal duct of *Lgr5* ^*+/+*^ **(I)** and the atypically formed nasopharyngeal duct of *Lgr5* ^*−/−*^ embryos **(J)**. The caudal area of the nasopharyngeal duct in WT embryo **(K)** and mutant embryo **(L)**. **(M–N)** Sagittal sections through the head display tongue fusion with mouth floor **(M´)** or dysregulation of salivary gland development **(N´)**. The expression of E-cadherin is not changed in *Lgr5* ^*+/+*^ **(O)** and *Lgr5* ^*−/−*^ **(P)**. Expression of β-catenin is decreased in developing nasopharyngeal duct area in *Lgr5* ^*−/−*^ in contrast to WT animal **(Q, R)**. Nuclei are counterstained by DAPI (Blue). e, eye; lg, lingual groove; Mc, Meckel’s cartilage; nd, nasopharyngeal duct; oc, oral cavity; p, palate; sg, salivary gland; t, tongue; vno, vomeronasal organ. Scale bars for A, B. G, H, M, N μ 1 mm. Scale bars for others = 100 μm.

**FIGURE 11:**
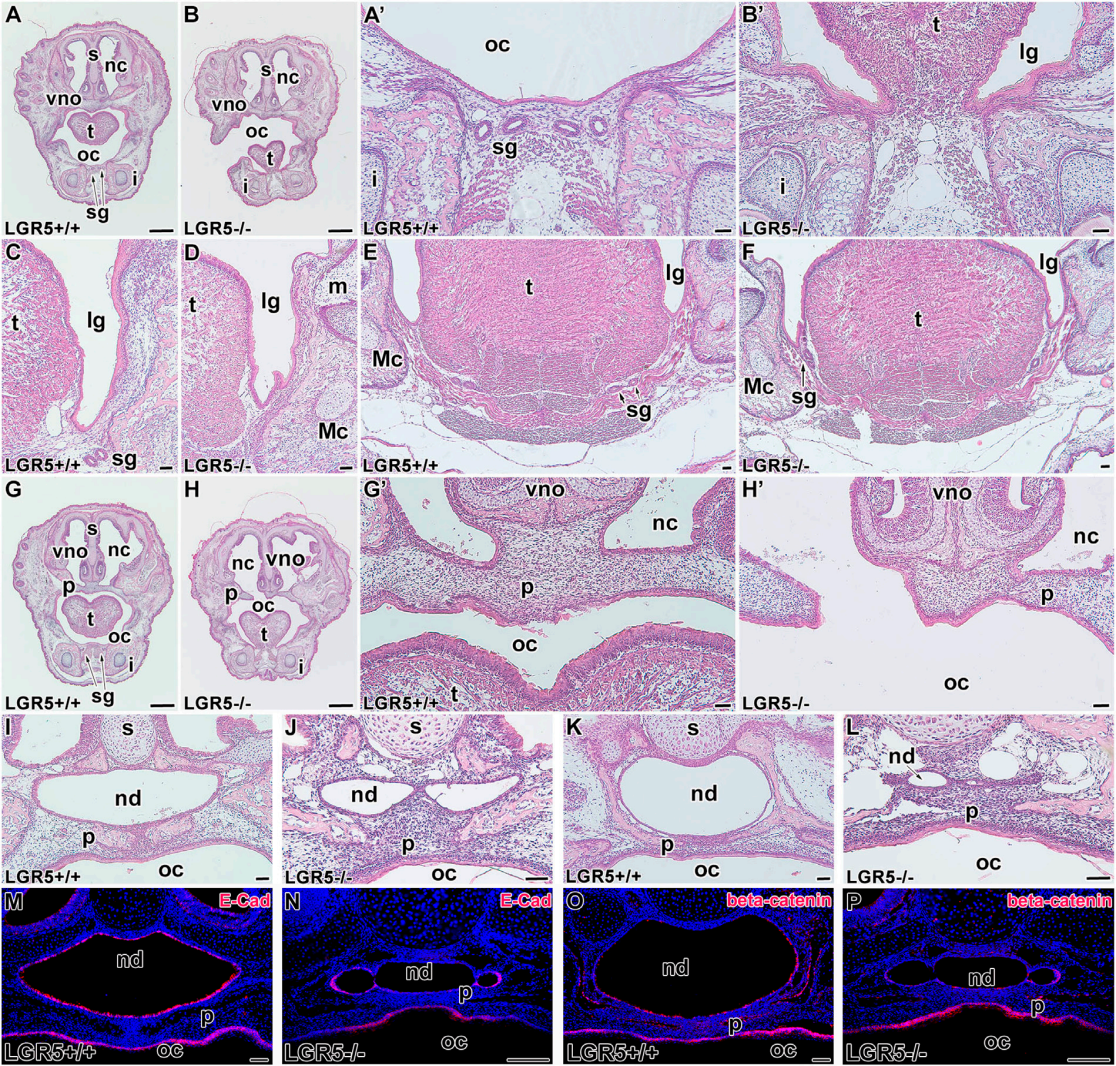
Craniofacial abnormalities of *Lgr5* ^*−/−*^ mice at E18.5. *Lgr5* ^*−/−*^ mice exhibit several craniofacial abnormalities also later in development at E18.5 **(A-L)**. Low-magnification images of transversal section through rostral area of the head in *Lgr5* ^*+/+*^ **(A)** and *Lgr5* ^*−/−*^ **(B)** mice stained by Hematoxylin-Eosin. Higher-magnification images of tongue area in WT embryo **(A´)** without abnormalities and *Lgr5*-deficient embryo with ankyloglossia **(B´)**. While there is not any defect in the area of lingual groove of WT embryo **(C,E)**, *Lgr5*-deficient mice exhibit noticeable epithelial branching and folding in the area of lingual groove **(D)**. Salivary glands are located in more caudal part in *Lgr5* ^*−/−*^ animals **(F)** in comparison to *Lgr5* ^*+/+*^ embryos **(E)**. Low magnification **(G)** and high magnification **(G´)** image of developing palate. In *Lgr5*-deficient mice, there is the cleft palate in low-magnification **(H)** as well as high-magnification **(H´)** images. **(J)** The histological sections of *Lgr5* ^*+/+*^ nasopharyngeal duct **(I)** and *Lgr5* ^*−/−*^ nasopharyngeal duct with distinct midline epithelial bridge. Caudal area of nasopharyngeal duct of WT embryo **(K)** and mutant embryo with distinct abnormalities such as an epithelial fusion of nasopharyngeal duct **(L)**. There is downregulation of E-cadherin expression mutant mice **(N)** in comparison to WT **(M)**. Also β-catenin expression is decreased in *Lgr5* ^*+/+*^ **(O)** and *Lgr5* ^*−/−*^ **(P)** mice. i, incisor; lg, lingual groove; m, molar; Mc, Meckel’s cartilage; nc, nasal cavity; nd, nasopharyngeal duct; oc, oral cavity; p, palate; s, septum; sg, salivary gland, t. tongue; vno, vomeronasal organ. Scale bars for A, B. G, H μ 1 mm. Scale bars for others = 100 μm.

To further investigate the role of LGR5 in the craniofacial structures, we generated *Lgr5*-deficient mice and analysed them at E16.5 (Figure 10) and E18.5 (Figure 11). Consistent with a previous study (Morita et al., 2004), we observed changes in the lingual groove area in both embryonic stages, where tongue ankylosis occurred in comparison with WT embryos (Figures 10A-B´, M, M´; Figures 11A-B´). In more caudal areas, there was a shallow lingual groove with epithelial folding at its base (Figures 10C, D, N, N´; Figures 11C, D). In addition, salivary glands were not visible rostrally or caudally, and the gland protrusions displayed altered patterning (Figures 10E-F´, N´; Figures 11E-F´). Changes in the palatal shelves shape resulted in a high palate with a groove in its ventral area, which was accompanied by a alterations in the intramembranous bone patterning in the palatal region and a delay in palatal seam disruption (Figures 10G-H´; Figures 11G-H´).

Defects in palatogenesis were associated with the reduction of the nasopharyngeal duct (Figures 10I-L; Figures 11I-L). The nasopharyngeal duct reduction was observed in all *Lgr5*-deficient animals either as a narrowing of the duct (Figures 10J, L; Figure 11L) or as a division of the duct into two channels by the formation of the midline epithelial bridge. The latter phenotype resulted in a full loss of communication with the nasopharynx (Figure 10J; Figure 11J). In the vomeronasal area, we observed a mild phenotype consisting of the changes in VNO orientation with respect to the nasal cavity (Figures 10A, B; Figures 11A, B). To confirm that the observed changes are related to loss of the LGR5 function and not caused by the absence of LGR5^+^ cells, we performed EGFP labelling on sections obtained from WT and *Lgr5* KO mice. The labelling demonstrated that cells transcribing the *Lgr5* locus are still present in affected structures in *Lgr5*-deficient animals (Supplementary Figure S7).

As we observed changes in epithelial structures, we followed them with analyses of key molecules responsible for their intercellular contacts and adhesions. The expression of E-cadherin was maintained in cell membranes of *Lgr5*-deficient animals at E16.5 (Figure 10P) although the cells were disorganized without their elongation and apparent polarization. At E18.5, strong downregulation of E-cadherin expression was observed, especially in the dorsal region (Figures 11M, N).

We further evaluated the membranous expression of β-catenin, the key mediator of canonical Wnt signalling (Wodarz and Nusse 1998) and a component of a protein complex that constitutes adherens junctions (Lewis et al., 1997). β-catenin expression was robust in elongated and polarised cells lining the nasopharyngeal duct, especially in apical parts of epithelial cells (Figure 10Q; Figure 11O). *Lgr5*-deficient animals displayed downregulation of β-catenin in this area with an unorganised expression pattern (Figure 10R; Figure 11P), indicating the release of intercellular connections contributing to defects during the modelling of epithelial structures.

### Effect of *Lgr5*-deficiency on Wnt signalling in craniofacial structures

**FIGURE 12:**
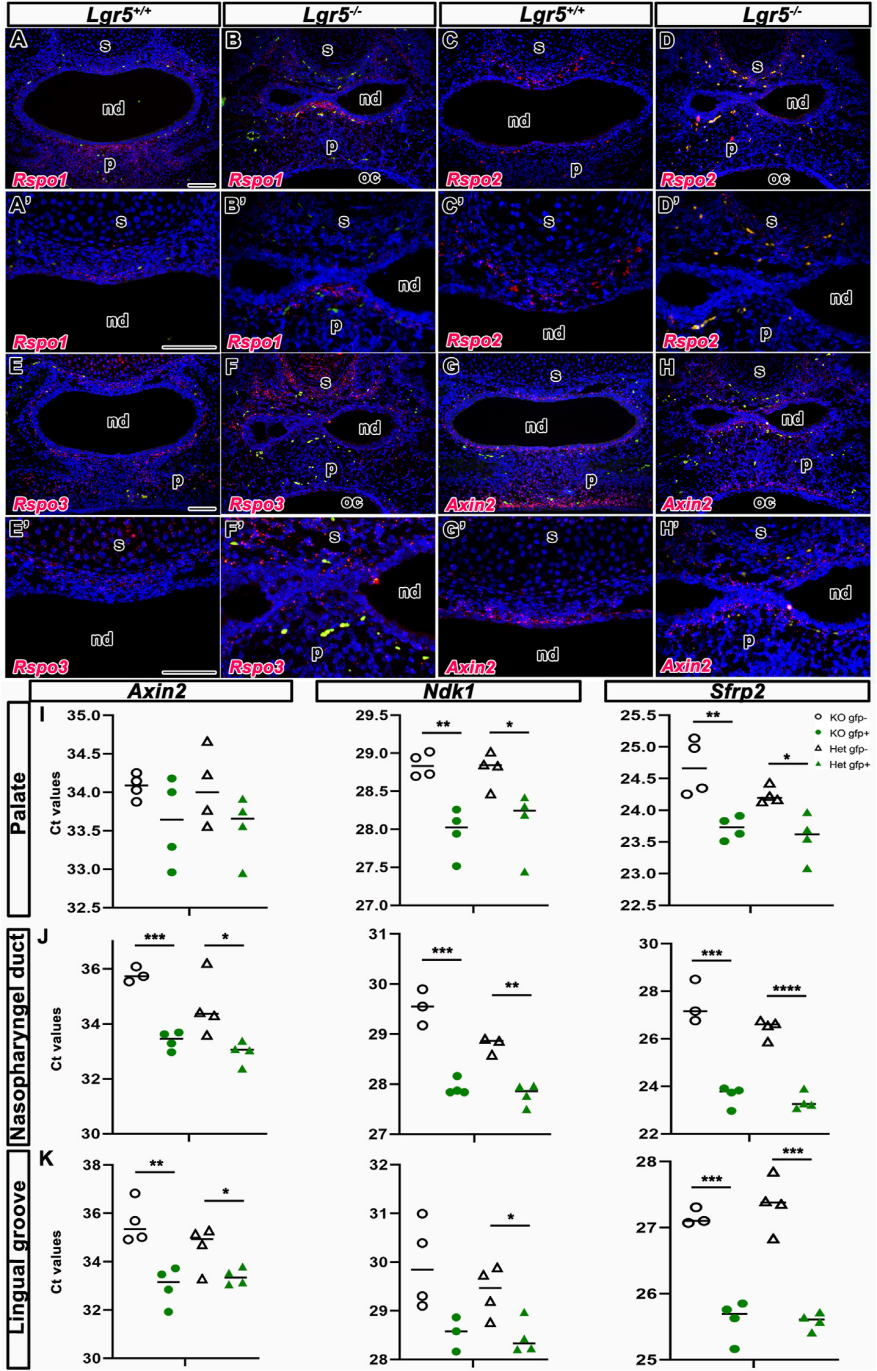
Wnt signaling in *Lgr5*-deficient mice. RNA expression of three LGR5 ligands—*Rspo1, Rspo2* and *Rspo3* and Wnt target gene *Axin2* in *Lgr5* ^*+/+*^ and *Lgr5* ^*−/−*^ mice at E16.5. The expression of genes is labelled by red color. Erythrocytes, which are naturally autofluorescent, are labelled by green color. RNA expression of *Rspo1* is observed in the mesenchyme surrounding nasal septum and nasopharyngeal duct. The significant expression of *Rspo1* is situated in palate **(A,A´)** of *Lgr5* ^*+/+*^ mice. The similar expression of *Rspo1* is observed in *Lgr5* ^*−/−*^ mice **(B,B´)**. *Rspo2* is expressed also in the mesenchyme surrounding nasal septum in both WT embryo **(C,C´)** and *Lgr5*-deficient mice **(D,D´)**. The expression of *Rspo3* is situated in the mesenchyme around nasopharyngeal duct and surrounding nasal septum in *Lgr5* ^*+/+*^ mice **(E,E´)** and similar expression pattern of *Rspo3* is found in *Lgr5* ^*−/−*^ mice **(F,F´)**, but it seems to be more disperse in the mesenchyme of *Lgr5* ^*−/−*^ embryo than *Lgr5* ^*+/+*^ embryo. *Axin2* expression is situated in the mesenchyme surrounding nasopharyngeal duct and nasal septum in both WT **(G,G´)** and mutant embryos **(H,H´)**. **(I-J)** qPCR analyses of selected Wnt genes in WT and KO mice embryos and in different craniofacial tissues. **(I)** The expression of *Axin2, Ndk1* and *Sfrp2* in the palate. The expression of all genes is higher in *Lgr5* ^*+/−*^ palatal cells in comparison to *Lgr5* ^*−/−*^ cells in both WT and mutant embryos. **(J)** In the area of nasopharyngeal duct, the expression of *Axin2, Ndk1* and *Sfrp2* is also increased in *Lgr5* ^*+/−*^ in comparison to cells collected from *Lgr5* ^*−/−*^ mice. **(K)** In the lingual groove, the expression of all genes is increased in *Lgr5* ^*+/−*^ cells in comparison to cells collected from *Lgr5* ^*−/−*^ animals. nd, nasophraryngeal duct; oc, oral cavity; p, palate; s, septum. Scale bars = 100 μm. The graphs indicate all values and average and they were compared to corresponding controls by *t*-test (unpaired, 2-tails) *p < 0.05; ** 0.05 < *p* < 0.01; ***p < 0.001; ****p < 0.0001.

To assess in Wnt signalling in affected areas of *Lgr5*-deficient animals, we first analysed the expression of different members of the Wnt pathway in craniofacial structures and evaluated their possible overlap in these areas with *Lgr5.* We assessed the possible alterations of their expression pattern in *Lgr5*-deficient animals. We selected R-spondins (Rspo), well-known LGR5 ligands and activators of Wnt signalling (Nagano 2019), and the target gene of the Wnt pathway *Axin2* (Jho et al., 2002) (Figure 12).

First, we analysed RNA expression of *Rspo1* in WT and *Lgr5* mutants at E16.5. The expression of *Rspo1* was observed in the mesenchyme underlying the epithelium of the nasopharyngeal duct and in the palatal area in both WT (Figure 12A) and *Lgr5-*deficient embryos (Figure 12B). We also found a strong expression of *Rspo1* in the mesenchyme surrounding the nasopharyngeal duct (Figures 12A, A´, B, B´). No changes in expression of *Rspo1* were observed when comparing WT and *Lgr5*-deficient embryos. Double labelling of *Lgr5* and *Rspo1* in WT embryos revealed cells expressing both molecules in the mesenchyme surrounding the nasopharyngeal duct (Supplementary Figures S8A, A´).

In addition, the expression of *Rspo2* was also only found in the mesenchyme surrounding the nasopharyngeal duct, close to the nasal septum in both WT (Figures 12C, C´) and *Lgr5* mutant embryos (Figures 12D, D´ in detail). The expression of *Rspo2* was more restricted to the mesenchyme surrounding the nasopharyngeal duct compared to the expression of *Rspo1* (Figures 12A-D´). *Lgr5* and *Rspo2* were co-expressed in the mesenchyme of the nasopharyngeal duct (Supplementary Figures S8B, B´).

The expression of *Rspo3* was located in the mesenchyme underlying the nasopharyngeal duct and close to the nasal septum in WT (Figures 12E, E´) and *Lgr5*-deficient animals (Figures 12F, F´). The expression of *Rspo3* was primarily dispersed in the mesenchyme of the nasopharyngeal duct of *Lgr5* mutant embryos compared to the expression of WT (Figures 12E, F). In addition, *Rspo3*-positive cells were observed in the cartilage of the nasal septum in WT (Figure 12E) and *Lgr5*-deficient animals (Figure 12F). The *Rspo3* signal was also more dispersed in the nasal mesenchyme when compared with *Rspo1* and *Rspo2* (Figures 12A-D´ compare to Figures 12E-F´).

An analysis of *Axin2* expression as a target gene of the Wnt pathway revealed *Axin2*-positive cells in the mesenchyme underlying the nasopharyngeal duct and palate in WT (Figures 12G, G´) as well as in *Lgr5* mutants (Figures 12H, H´). When we analysed the co-expression of *Lgr5* and *Axin2,* we uncovered an overlap of these Wnt target genes in the mesenchyme surrounding the nasopharyngeal duct (Supplementary Figures S8C, C´).

To investigatethe impact of *Lgr5*-deficiency on canonical Wnt signalling in the craniofacial structures, we collected samples from the lingual groove area, palate, and roof of the nasopharyngeal duct of mice at stage E16.5. Using *Lgr5-EGFP-CreERT2* mice, we sorted LGR5/EGFP-positive and LGR5/EGFP-negative cells according to the activity of the endogenous Lgr5 promoter to screen them separately for Wnt target genes (Figures 12I–K). We compared the expressions of the Wnt target genes *Axin2*, Naked Cuticle Homolog 1 (*Nkd1*), and Secreted Frizzled Related Protein 2 (*Sfrp2*). Interestingly, there was no change in the expression of all three genes studied in LGR5/EGFP-positive cells sorted from *Lgr5*-deficient and *Lgr5*-heterozygous animals. However, in LGR5/EGFP-negative cells from *Lgr5* ^*−/−*^ animals, the expression of Wnt target genes was only slightly downregulated compared with *Lgr5* ^*+/−*^ embryos.

The only significant change we uncovered was an increased expression of the studied Wnt target genes in the EGFP-positive population compared to EGFP-negative cells. The effect was strongest in the nasopharyngeal duct region (Figure 12J). Our results suggest that mesenchymal cells in the craniofacial region exhibit active Wnt signaling independent of *Lgr5* gene status.

### Gene expression profile of LGR5-positive cells

Because we observed only small differences in the gene expression of Wnt target genes between LGR5-positive and LGR5-negative cell populations in *Lgr5* ^*−/−*^ and *Lgr5* ^*+/−*^ embryos, we next asked what other differences might be found in gene expression of additional types of Wnt signalling pathways or whether LGR5 in craniofacial mesenchyme might not play a role in completely different, i.e. Wnt signaling-independent signalling events that were originally proposed for gut epithelial cells.

**FIGURE 13:**
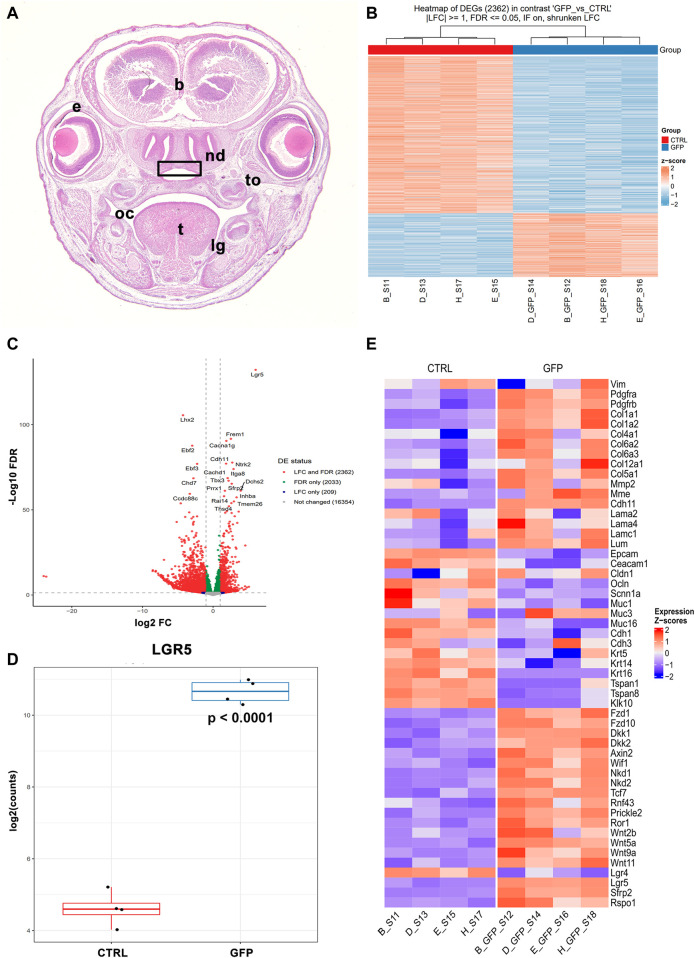
Gene expression profile of LGR5-positive and LGR5-negative cells by RNA sequencing. **(A)** Roof of nasopharyngeal area (rectangle) was collected from animals *Lgr5* ^*+/−*^ mice at E16.5 as shown of transversal section through the head stained by Hematoxylin-Eosin. **(B)** LGR5/EGFP-positive and LGR5/EGFP-negative cells were sorted and analyzed separately. Four samples were used for each cell populations and these samples exhibit high similarity as shown on heat map. 2362 genes exhibited statistically significance between groups. **(C)** Volcano plot of significantly expressed genes. **(D)** Differences in expression of *Lgr5* in LGR5/EGFP-positive and LGR5/EGFP-negative cells. **(E)** Heat map of feature genes with focus on epithelial and mesenchymal markers as well as Wnt signaling pathway members. The graphs values indicate average ±SD and *p* value when data were compared with the corresponding control group.

We again used sorted EGFP-positive and EGFP-negative cells from previous analyses obtained from the roof of nasopharyngeal duct of *Lgr5* ^*+/−*^ mice at stage E16.5 (Figure 13A), whereas we used *Lgr5-EGFP-CreERT2* mice. The roof of the nasopharyngeal duct was selected for RNAseq analyses because the expression of LGR5 was high at E16.5, and we had already observed some statistically significant gene expression changes by qRT-PCR. Four samples were analysed for EGFP-positive and EGFP-negative cells. Distinct gene expression profiles were found between these 2 cell populations (Figure 13B), where 2362 genes displayed statistically significant differences in their expression. As expected, the greatest difference was found in the expression of *Lgr5* (Figures 13C, D), confirming our successful sorting of these cells, even it was performed based on their EGFP expression.

In the LGR5-negative population, we detected a high expression of epithelial markers such as Epithelial Cell Adhesion Molecule (*Epcam*)*,* Cadherin 1 (*Cdh1*)*,* Keratin 14 (*Krt14*)*,* Claudin 9 (*Cldn9*)*,* and Mucin 16, Cell Surface Associated (*Muc16*) (Figure 13E; Supplementary Figures S9A-D). LGR5/EGFP-positive cells, on the other hand, were characterised by the expression of several mesenchymal markers such as Vimentin (*Vim*), numerous collagen species, Matrix Metallopeptidase 2 (*Mmp2*)*,* Twist Family BHLH Transcription Factor 1 (*Twist1*) and many others (Figure 13E; Supplementary Figures S9E-H). These findings further supported our previous histological analyses for the preferential expression of *Lgr5* in the mesenchymal cells of craniofacial areas.

Next, we investigated the possible differences in gene expression between LGR5/EGFP-positive and LGR5/EGFP-negative cells in relation to the status of the canonical Wnt signalling (Figure 13; Supplementary Figure S10). Interestingly, many target genes (Nusse 2022) of Wnt pathway, including *Axin2*, Dickkopf Wnt Signaling Pathway Inhibitor 1 (*Dkk1*), *Nkd1*, Ring Finger Protein 43 (*Rnf43*), *Sfrp2*, Transcription Factor 7 (*Tcf7*), and Wnt Inhibitory Factor 1 (*Wif1*), were found upregulated in LGR5-positive cells and non-canonical pathway components such as Receptor Tyrosine Kinase Like Orphan Receptor 1 (*Ror1*)*,* Prickle Planar Cell Polarity Protein 2 (*Prickle2*)*,* Wnt Family Member 5A (*Wnt5a*)*,* Wnt Family Member 11 (*Wnt11*)*,* Frizzled Class Receptor 10 (*Fzd10*) were found to be upregulated in LGR5-positive cells (Figure 13; Supplementary Figure S10). A few Wnt components, such as Wnt Family Member 7B (*Wnt7b*)*,* Wnt Family Member 10A (*Wnt10a*)*,* Frizzled Class Receptor 9 (*Fzd9*)*,* FOS Like 1, AP-1 Transcription Factor Subunit (*FoslI*)*,* Junction Plakoglobin (*Jup*), were downregulated with MAP pathway components such as Rac Family Small GTPase 2 (*Rac2*) or Mitogen-Activated Protein Kinase 10 (*MAPK10*) and calcium signalling (Calcium/Calmodulin Dependent Protein Kinase II Beta, *Camk2b*).

### LGR5-positive cells express numerous molecules associated with cell adhesion and components of the basal lamina

**FIGURE 14:**
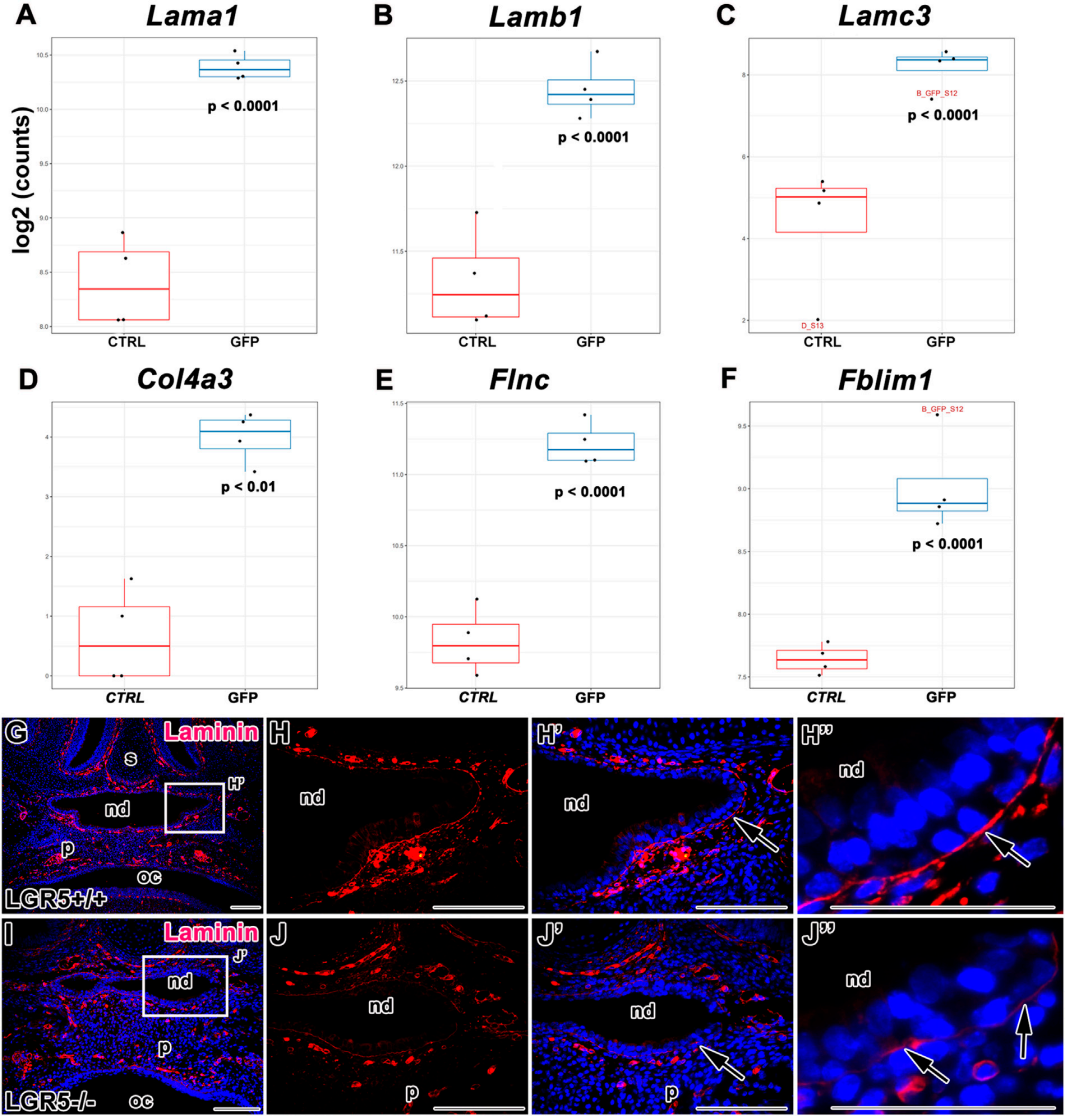
Gene expression of molecules associated with basal membrane. **(A–F)** Differences in gene expression of *Lama1, Lamb1, Lamc3, Flnc* and *Fblim1* in LGR5/EGFP-positive and LGR5/EGFP-negative cells. Laminin expression in WT **(G-H´´)** and *Lgr5*-deficient animals **(I-J´´)** detected by immunohistochemistry displays weaker signal of basal membrane marker in mutant animals nd, nasopharyngeal duct; oc, oral cavity; p, palate; s, septum. Scale bars = 100 μm. The graphs values indicate average ±SD and *p* value when data were compared with the corresponding control group.

Interestingly, the RNA-seq data uncovered alterations in the gene expression of numerous components involved in the interaction with the extracellular matrix (Figures 13, 14). Molecules associated with the basal membrane such as Laminin Subunit Alpha 1 (*Lama1*)*,* Laminin Subunit Beta 1 (*Lamb1*), Laminin Subunit Gamma 3 (*Lamc3*) and Collagen Type IV Alpha (*Col4a*), were upregulated in LGR5-positive cells (Figures 14A–D). In addition, we determined higher expression of Filamin (*Flnc*), a key molecule for interaction with actin filaments (Figure 14E) and anchoring the membrane proteins to the actin cytoskeleton, or Filamin Binding LIM Protein 1 (*Fblim1*), which is important for cell junctions and connection of cell adhesion structures to the actin cytoskeleton, was increased in LGR5-positive cells (Figure 14F).

Because of the robust differences in basal membrane components expression between LGR5-positive and LGR5-negative cells, we next tested whether there were any changes in the basal membrane structure in *Lgr5*-deficient mice. Indeed, we found distinct differences in the laminin expression in the basal lamina of the epithelium surrounding the nasopharyngeal duct in mutant embryos (Figures 14I-J´´) in contrast to WT embryos (Figures 14G-H´´). Laminin expression displayed a disorganized pattern in *Lgr5*-deficient animals. In this region of disordered epithelium of the nasopharyngeal duct (Figure 14J´´), we observed the disappearance of laminin expression in *Lgr5-*mutated embryos. Whereas laminin expression was strong and continuous in WT embryos (Figure 14H´´).

## Discussion

### LGR5 expression in the mesenchyme of craniofacial structures

LGR5 is known as a marker of stem and progenitor cells in many epithelial tissues, such as the intestine (Barker et al., 2007), stomach (Barker et al., 2010), hair follicles (Haegebarth and Clevers 2009), mammary glands (De Visser et al., 2012), kidneys (Barker et al., 2013), and inner ear (Bramhall et al., 2014). However, up to now, only a few studies have focused on the expression and function of LGR5 during craniofacial development, especially in structures such as a palate or VNO. Most available information about *Lgr5* expression during craniofacial development concerns the incisor and molar region (Kawasaki et al., 2014) or the tongue area (Morita et al., 2004; Yee et al., 2013). Our analysis revealed a similar distribution of LGR5-positive cells in the tongue and lower jaw as was previously published (Morita et al., 2004). Additionally, we detected LGR5 expression in the mesenchyme of the developing lingual groove, which can also be associated with the phenotype of *Lgr5*-deficient mice.

In the upper jaw, a distinct expression of *Lgr5* has been previously found in neural crest cells from E9.5 and the adult oral mesenchymal cells derived from these cells (Boddupally et al., 2016). Recently, *Lgr5* expression was found in the area undergoing lip fusion and during eye development from E10.5 to E13.5 (Nihara et al., 2021). Here, we further uncovered distinct LGR5 expression in the palatal area during palatal shelf closure, the associated nasopharyngeal duct, during VNO development and nasal cavity formation. In all these areas, LGR5-positive cells were located in the mesenchyme adjacent to the epithelium undergoing folding. A decrease in LGR5-positive cell numbers was observed with increasing embryonic age, and a very low signal was found at the postnatal stage. Interestingly, all newly described LGR5-positive areas correspond to structures exhibiting developmental defects in *Lgr5*-deficient embryos.

While LGR5-positive cells are typically described as preferentially located in the epithelia in other organ systems, epithelial expression of LGR5 is unusual in craniofacial structures and the majority of LGR5-positive cells were found in the craniofacial mesenchyme. Interestingly, a population of LGR5-positive mesenchymal cells called telocytes have recently been found in the intestine (Bahar Halpern et al., 2020). LGR5-positive mesenchymal cells closely resembling telocytes can also be found in the mammary gland, muscles, and lung (Wang et al., 2015; Lee et al., 2017; Leung et al., 2020). It is still unclear why there is a preference for LGR5 expression in the craniofacial region to the mesenchyme instead of epithelium, which will be a topic for future studies.

### Impact of LGR5 deficiency on craniofacial development

Deficiency in *Lgr5* causes lethality in neonatal mice because of the inability to suck the milk (Morita et al., 2004). *Lgr5*-deficient mice have a defective separation of the tongue from the mandibular prominence leading to the fusion of the ventral part of the tongue and the floor of the oral cavity. This malformation, called ankyloglossia, is accompanied by gastrointestinal tract dilatations (Morita et al., 2004) and corresponds to a higher expression of LGR5 on the labial side of the developing lingual groove. However, no additional craniofacial phenotypes associated with *Lgr5* gene inactivation have been described up to now in this animal model despite of fact that *Lgr5* is expressed in several localised and large domains in the craniofacial mesenchyme. Therefore, we generated *Lgr5*-deficient mice to analyse the less severe craniofacial defects caused by *Lgr5*-deficiency.

Since the animals survived to birth, we could also focus on the late foetal stages when organogenesis occurs. At both our analysed stages (E16.5 and E18.5), we found ankyloglossia similar to previously published data (Morita et al., 2004). Surprisingly, the direct sequencing of the *Lgr5* gene in humans with ankyloglossia did not reveal any mutation in this gene (Acevedo et al., 2010; Lenormand et al., 2018). However, whether patients with an *LGR5* mutation have disorders associated to the tongue development still needs to be investigated in detail.

In *Lgr5*-deficient mice, we uncovered additional changes in the caudal area of the tongue, such as folding of the base of the lingual groove and an increased number of salivary gland ducts. On the other hand, no gland ducts were observed in the jaw’s rostral area, which contrasts with the arrangement of salivary ducts in WT animals. Previously, *Lgr5* expression was found in the primary duct of the developing submandibular gland and the surrounding mesenchyme. Nevertheless, it is not yet proven whether *Lgr5* expression labels a population of stem/progenitor cells in the large salivary glands of mice (reviewed in Emmerson and Knox 2018). Interestingly, LGR5-positive mesenchymal cells in human minor salivary glands displayed an ability of self-renewal and multipotency (Lu et al., 2015). However, whether LGR5-positive cells are important for salivary gland development is not yet known. Our analysis of *Lgr5* knockout mice implies this protein’s possible role in the interaction between mesenchymal cells and adjacent glandular epithelium in early gland development when the number of ducts is set up.

In *Lgr5*-deficient animals, we observed an abnormal shape of the oral roof, with the palatal shelves protruding dorsally after closure and forming a furrow in the midline region. Moreover, the reduction of the nasopharyngeal ducts occurred in association with the narrowing of the duct cavity, especially in the caudal area or its full closure, together with the loss of connection between the nasal cavity and the pharynx. Previously, no disruption of palatogenesis was observed in *Lgr5*-deficient animals (Morita et al., 2004). However, microscopic images from the palatal area were not discussed or provided in that study. Therefore, the phenotype occurring in the caudal region might be originally overlooked without evidence of macroscopic abnormalities observable from the palatal area. It should also be noted that a complete cleft palate was found in *Lgr5/6* double knockout and *Lgr4/5/6* triple knockout mice embryos (Szenker-Ravi et al., 2018); therefore, a deficiency of just *Lgr5* caused only a mild palatal phenotype. Our sequencing data revealed a high expression of *Lgr6* in LGR5-positive cells, suggesting that *Lgr5* deficiency is partially rescued by expression of *Lgr6* in the craniofacial region.

### Fate of mesenchymal LGR5-positive cells

LGR5-positive cells are located at the base of the intestinal crypts or in the deep part of the hair follicle bulge and can form the entire intestinal epithelium or renew the hair follicle (Haegebarth and Clevers, 2009). Like the intestine, LGR5^+^ stem/progenitor cells were found at the base of the circumvallate papilla of the tongue. Lineage tracing analysis revealed that these cells give rise to multiple cell types; they exhibit self-renewal and persistence at the base of the circumvallate papilla (Yee et al., 2013). LGR5-positive cells were also located in the ventral tongue stroma (involved in the stroma maintenance) and in some areas of the oral mucosa that originated from the neural crest (Boddupally et al., 2016). Due to their capacity for self-renewal, LGR5-expressing cells also play an important role in adult tissue repair. For example, in the taste buds, an LGR5-positive cell population was impaired after irradiation leading to a reduction in progenitor cell proliferation (Gaillard et al., 2019). In all these cases, LGR5-positive cells are multipotent stem cells capable of replacing different cell populations.

We followed the fate of LGR5-positive mesenchymal cells in several craniofacial structures during their development. In contrast to epithelial LGR5-positive cells, which produce distinct progeny, we observed only a few LGR5-positive cells in the craniofacial mesenchyme, including the palatal and VNO region, in the progeny. Using a lineage tracing approach, we found that most of the tdTomato-labeled cells retained LGR5 expression, i.e., the tdTomato-based red fluorescence overlapped with the EGFP signal. Double-positive, i.e., co-expressing tdTomato and EGFP, LGR5-positive cells were observed in the mesenchyme of the nasal septum and in the mesenchyme underlying the dorsal epithelium of the nasopharyngeal duct during palatogenesis, suggesting that they retain LGR5 expression during embryogenesis. Only a few tdTomato^+^ cells, i.e. cells originally LGR5-positive, lost their LGR5/EGFP expression and migrated to new areas primarily adjacent to the regions retaining LGR5-positive cells. These cells possibly underwent a phenotypic change/differentiation accompanied by downregulation of LGR5 expression. Interestingly, tdTomato-positive cells persisted in the craniofacial mesenchyme days up to the birth. Our observation suggests that LGR5-positive mesenchymal cells do not represent typical stem or progenitor cells. This finding is also supported by the fact that there was not found the overlap with other stem cell markers in epithelial tissues of the craniofacial region. Further experimental work is needed to uncover LGR5 function(s) in the mesenchymal cells during embryogenesis.

### Other possible roles of LGR5 in craniofacial development

Concerning cell non-autonomous effects, we documented a distinctive effect of *Lgr5* deficiency on the organisation of epithelial cells adjacent to LGR5-positive mesenchymal cells. We detected downregulation of E-cadherin and β-catenin expression in *Lgr5*-deficient embryos, which was associated with a clear disintegration of epithelial sheets and loss of the epithelial cell polarity in the lingual grove, adjacent salivary glands, and nasopharyngeal duct area. To explain these phenotypes, we propose a tight interplay between epithelium and the underlying LGR5-positive mesenchymal cells during craniofacial development contributing to epithelial morphogenesis. Similar interaction have been documented in many adult tissues, where mesenchymal cells provide crucial niche factors to epithelial cells. Our RNA sequencing of LGR5-positive cells from the nasopharyngeal duct area uncovered the expression of several molecules associated with the basal membrane, a key structure for epithelial morphogenesis. When we analysed laminin expression in *Lgr5*-deficient animals, we found it was downregulated in areas where epithelial cells were disorganised.

Moreover, alterations of *Lgr5* expression can affect the cytoskeletal structure of actin and cell adhesions through IQGAP1 (IQ Motif Containing GTPase Activating Protein 1) phosphorylation and its binding to Rac1 (Carmon et al., 2017). Elimination of *Lgr5* in colon cancer cells results to in reduced cell-cell adhesions. Similarly, RNA sequencing showed the expression of several molecules associated with actin arrangement in LGR5-positive cells in craniofacial structures. The downregulation of adhesion molecules was found in the epithelium adjacent to originally LGR5-positive mesenchyme in *Lgr5*-deficient animals. Based on these results, we propose that the absence of LGR5 leads to a release of intercellular contacts causing the disruption of epithelial fold formation. Therefore, LGR5-positive mesenchymal cells appear to play a key role in epithelial cell organisation and the final shaping of craniofacial structures. Similarly, RNA sequencing data from adult lungs provided clear evidence that *Lgr5*-positive mesenchymal cells support proper differentiation and organisation of epithelial cells by secreting Fibroblast Growth Factors (FGFs) and Wnt ligands in response to Wnt pathway activation (Kumar et al., 2014; Lee et al., 2017).

### Wnt signalling and LGR5 in the craniofacial area

LGR5 is an agonist of canonical and non-canonical Wnt signalling after RSPO binding (Glinka et al., 2011; Koo et al., 2012). In mesenchymal cells, Wnt signalling was implicated in the downregulation of E-cadherin, loss of epithelial signature, and the epithelia-mesenchymal transition during palatal shelves fusion (Nawshad and Hay 2003), and this phenomenon was confirmed in other tissues (Tan et al., 2001; Kim et al., 2002; Liebner et al., 2004; Mohamed et al., 2004; Nawshad et al., 2004). Because of these findings, we anticipated that higher levels of Wnt signalling could impose a mesenchymal phenotype of LGR5-positive cells to enable tissue remodelling close to the epithelial folds in the craniofacial area. However, when we analysed the expression of *Axin2*, which is considered one of the “universal” markers of canonical Wnt signalling (Kikuchi, 1999), *Axin2*-positive cells overlapped *Lgr5*-positive areas, especially in the mesenchyme surrounding the nasopharyngeal duct. Similarly, *Lgr5* gene expression analyses revealed some overlap with *Rspos* in several craniofacial areas, including the upper lip or eyelid areas (Nihara et al., 2021). Surprisingly, there were only minor changes or decreases in the expression of *Axin2* or *Rspo1-3* in *Lgr5*-deficient animals, suggesting a role for *Lgr5* as a Wnt agonist in *Lgr5*-expressing cells.

Alternatively, RSPO-LGR5 signalling could trigger a non-canonical branch of Wnt signalling in mesenchymal cells. Wnt/planar cell polarity (PCP) signalling is important to determine the polarity of cells in numerous tissues. In vertebrates, the Wnt/PCP pathway plays a role in the developmental processes such as the orientation of hair cells in the inner ear, narrowing the mediolateral axis in embryos, or extension of the anterior-posterior axis during gastrulation (Wansleeben and Meijlink 2011). Some co-expression of the Wnt/PCP pathway components and LGR5 in neural crest cells important for the development of craniofacial structures (Darken et al., 2002), was observed, and our RNA sequencing confirmed the expression of several members of noncanonical Wnt signalling in LGR5-positive cells. Although LGR5 has not been previously found to be an activator of the Wnt/PCP pathway during craniofacial development, it was shown to be able to mediate this signalling pathway during gastrulation (Glinka et al., 2011). Therefore, it is possible that LGR5 expressed in the mesenchymal cells of the developing grooves of the palate, eyelids, or tongue can be responsible for the polarity changes and cell rearrangement during fold formation. However, as suggested earlier, it is also possible that LGR5 functions in mesenchymal cells via an alternative mechanism independent of RSPO ligands and unrelated to Wnt signalling. An example would be the LGR5 paralog LGR4, which acts as an alternative receptor for Tumor Necrosis Factor (Ligand) Superfamily, Member 11 (TNFSF11; also known as RANKL) (Luo et al., 2016).

## Conclusion

Here, we analyzed the distribution of LGR5-positive cells in different craniofacial structures during embryonic development, focusing on the palate, lingual groove, and vomeronasal organ. Surprisingly, LGR5-positive cells were found mainly in the craniofacial mesenchyme, although the function of LGR5 was previously associated mainly with epithelial tissue. Lineage tracing and *Lgr5* gene knockout experiments have confirmed that LGR5-positive mesenchymal cells form a distinct subpopulation that is critical for proper craniofacial morphogenesis. Most of these cells are located in areas closely adjacent to specific epithelial regions, and they have limited migratory potential. Detailed analyses of *Lgr5*-deficient animals revealed several new phenotypic features in craniofacial structures including reduction or loss of connection between the nasal cavity and nasopharynx by abatement of the nasopharyngeal duct. Analysis of the expression profiles of mesenchymal cells labelled with EGFP expressed from the endogenous *Lgr5* locus uncovered that the LGR5 in these cells does not act as an agonist of the canonical Wnt signalling but likely plays a different role independent of the Wnt pathway. Thus, our analysis suggests possible novel functions of LGR5 during craniofacial development as a regulator of epithelial morphogenesis.

## Data Availability

The original contributions presented in the study are included in the article/Supplementary Material, further inquiries can be directed to the corresponding author.
