# Supplementary figures and images for: Role of LGR5-positive mesenchymal cells in craniofacial development
Source: Front Cell Dev Biol. 2022 Sep 5;10:810527. doi: 10.3389/fcell.2022.810527 (PMC9484000; doi:10.3389/fcell.2022.810527)

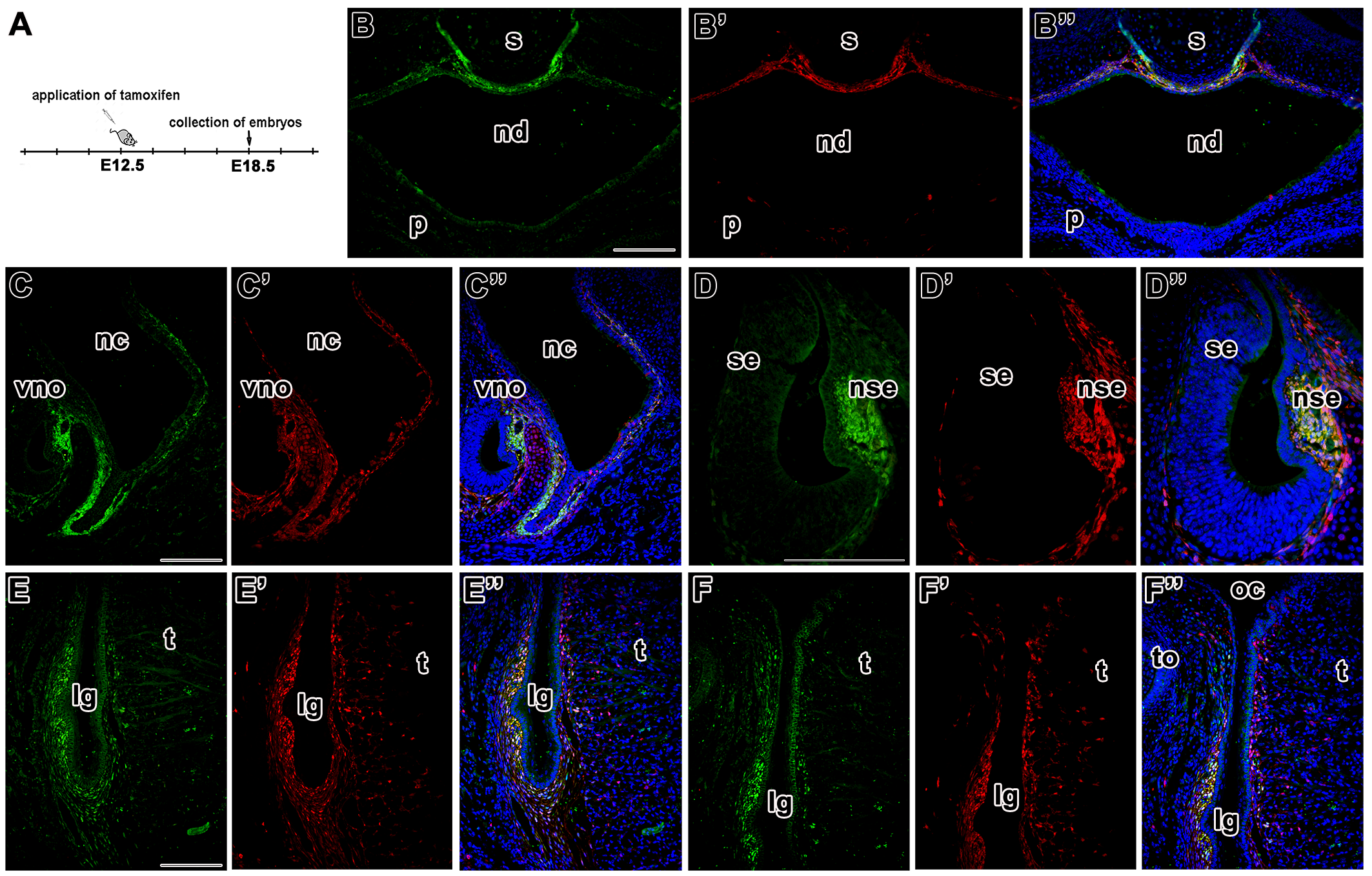

Supplement: Supplementary file 1 [file Image6.TIF]

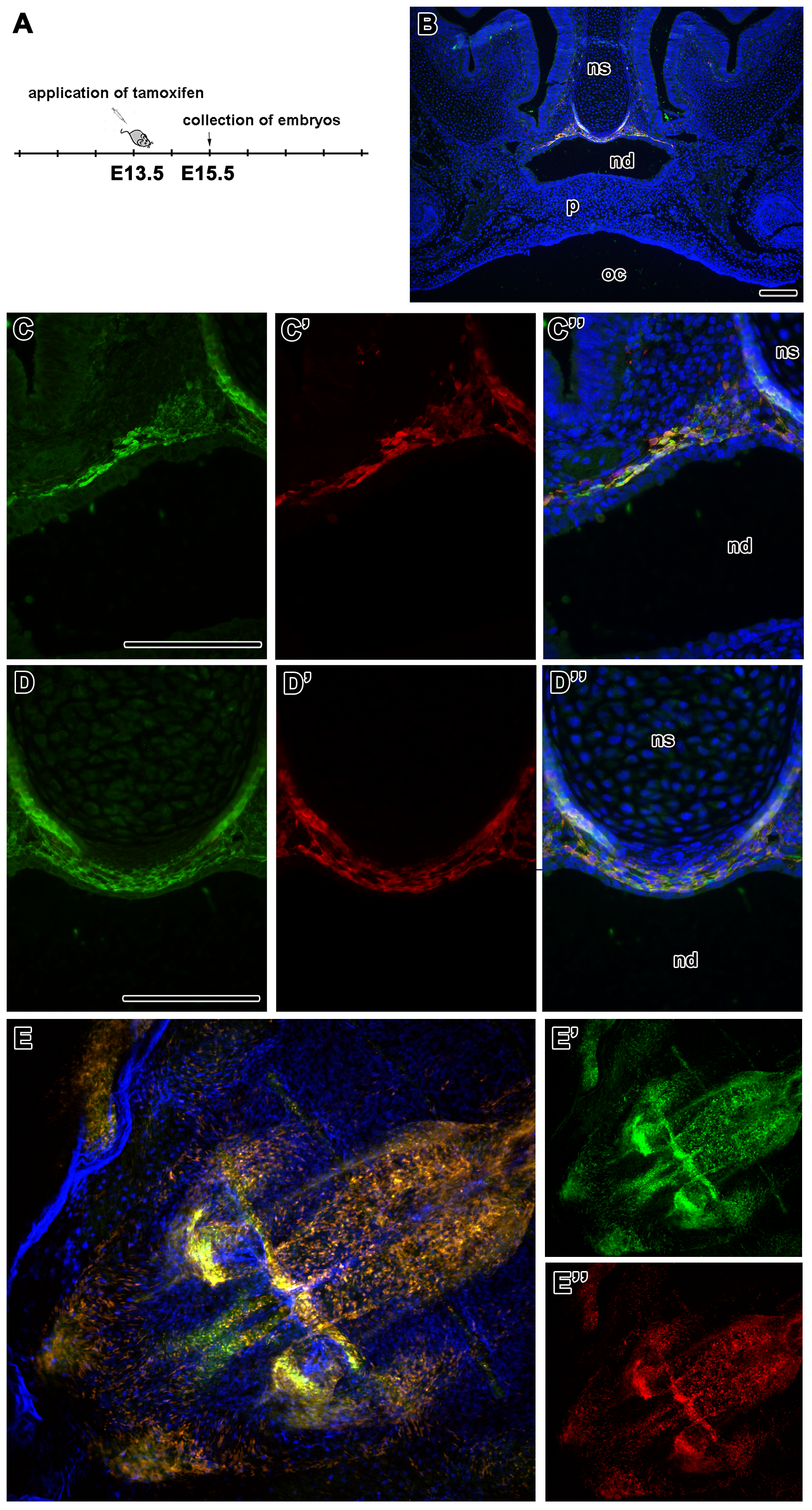

Supplement: Supplementary file 2 [file Image3.TIF]

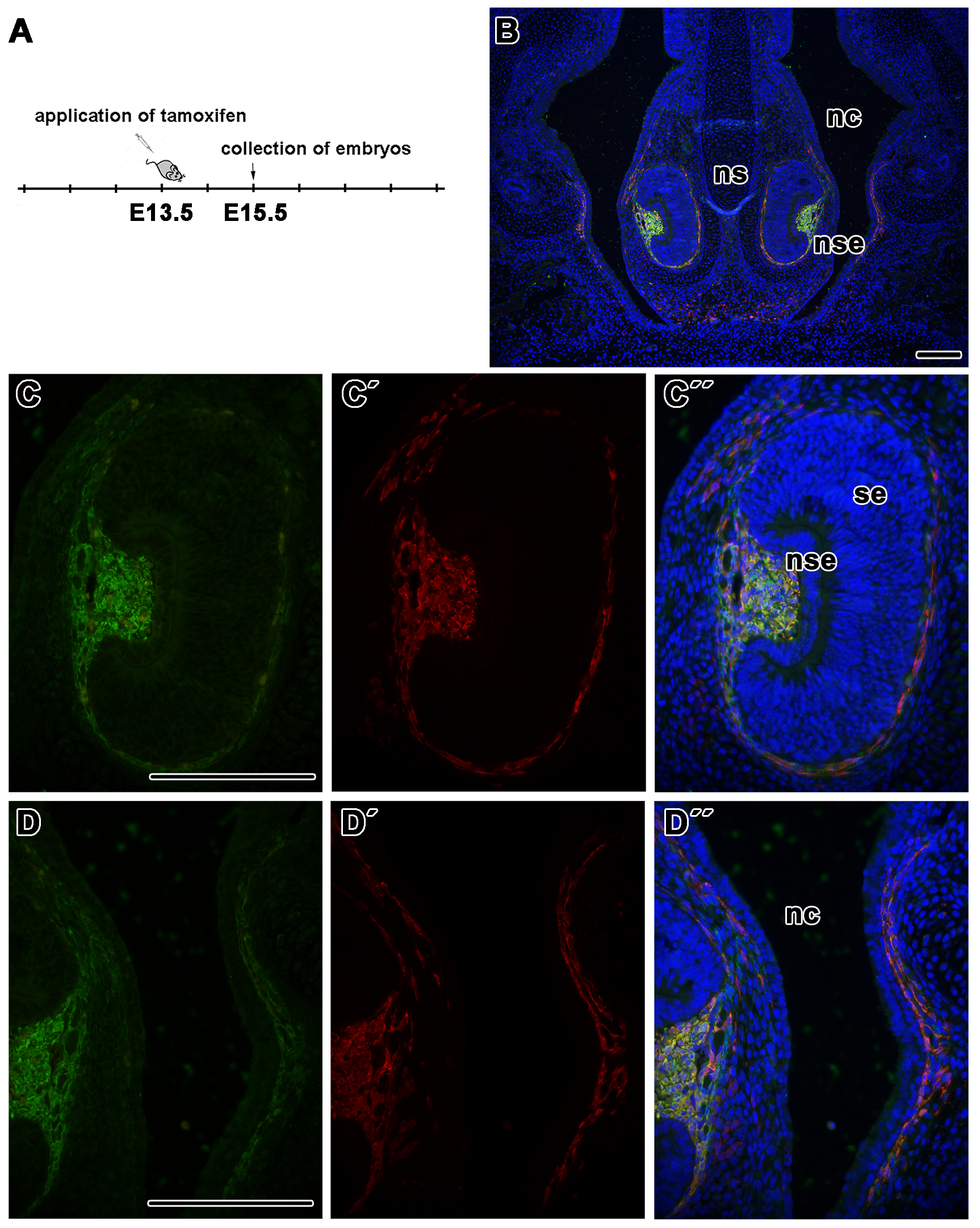

Supplement: Supplementary file 3 [file Image4.TIF]

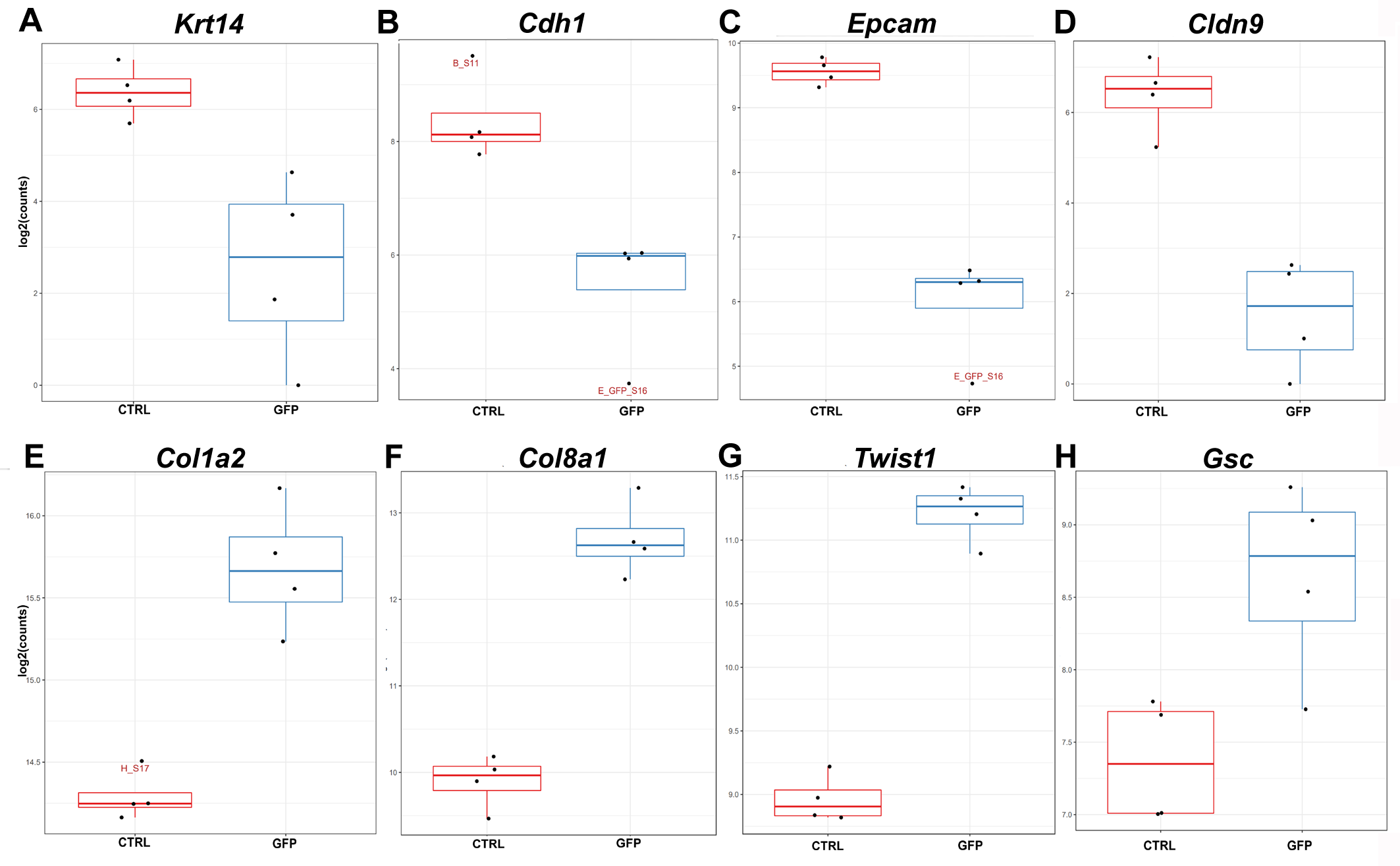

Supplement: Supplementary file 4 [file Image9.TIF]

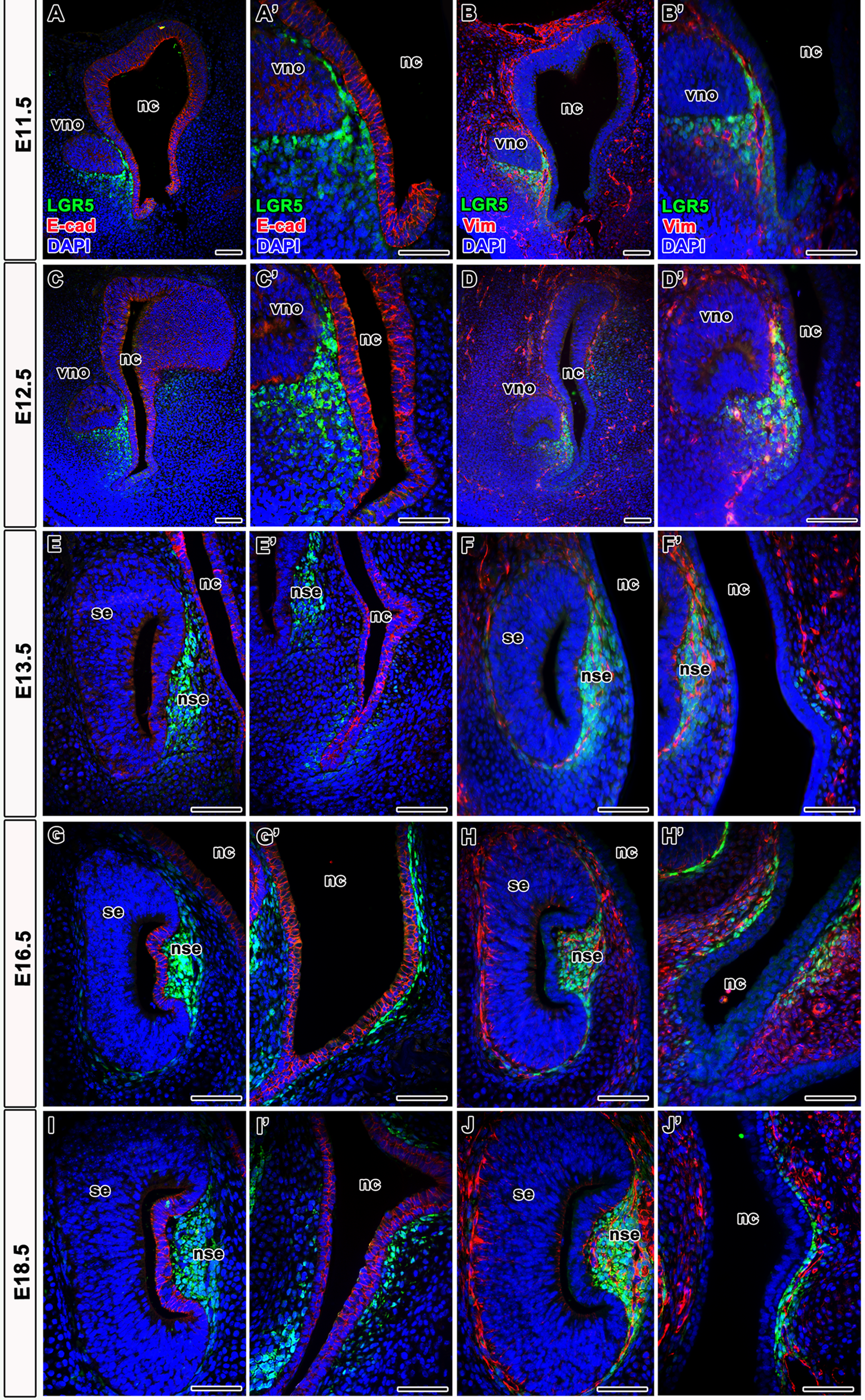

Supplement: Supplementary file 5 [file Image2.tif]

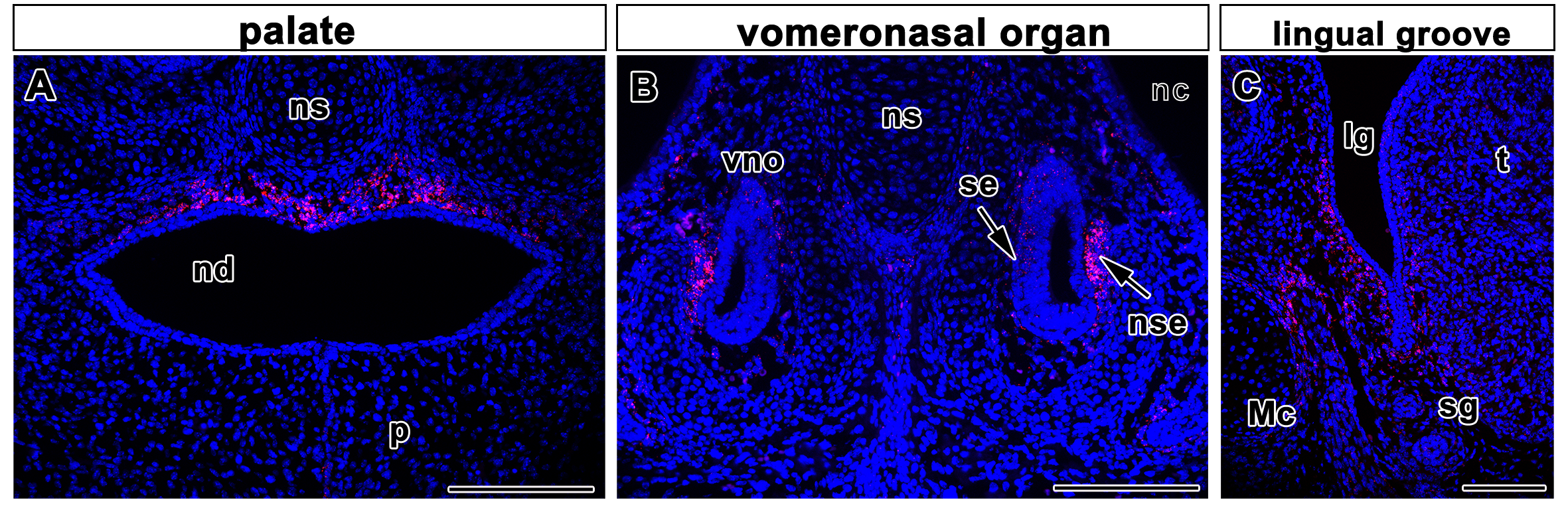

Supplement: Supplementary file 6 [file Image1.tif]

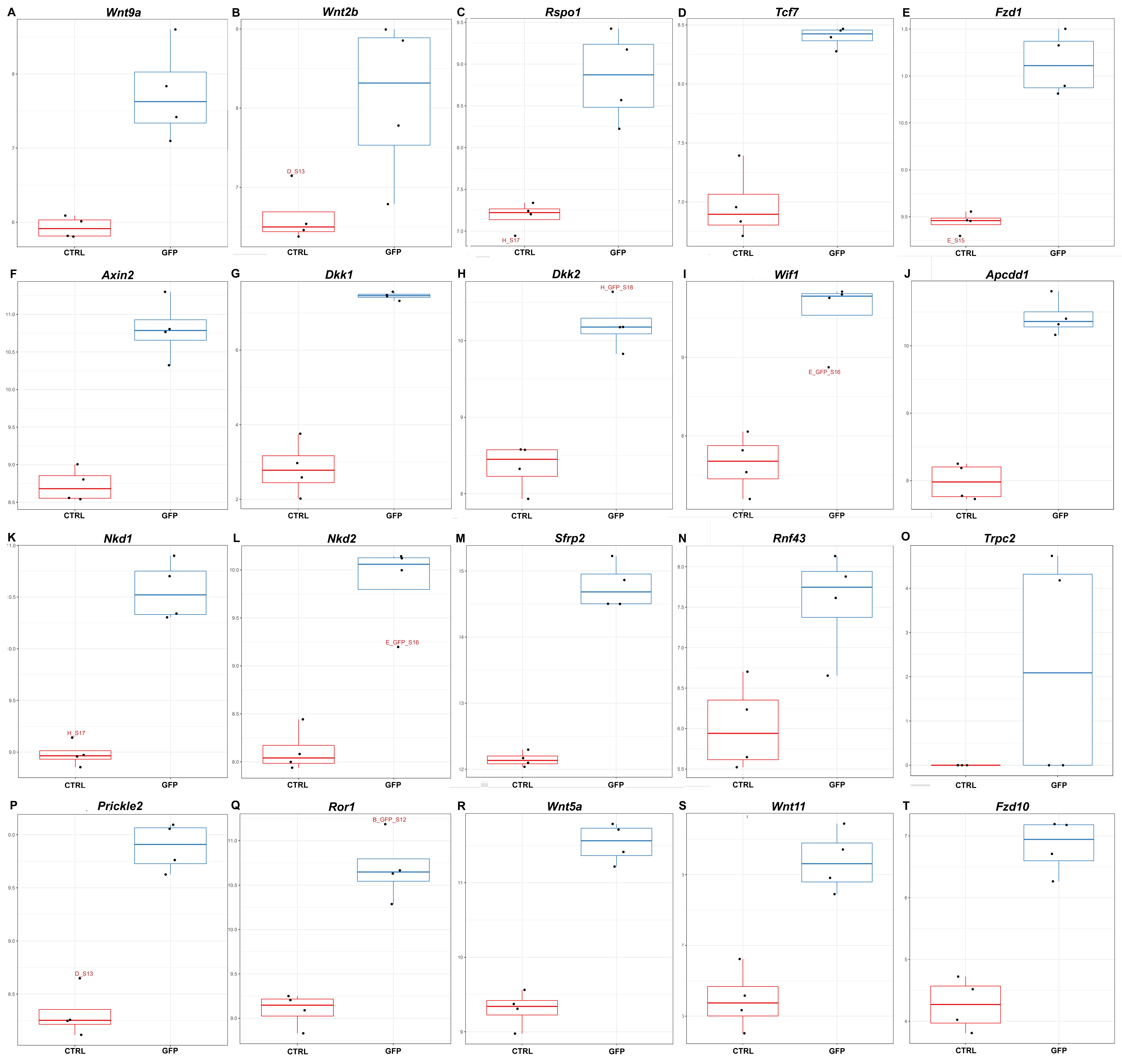

Supplement: Supplementary file 7 [file Image10.TIF]

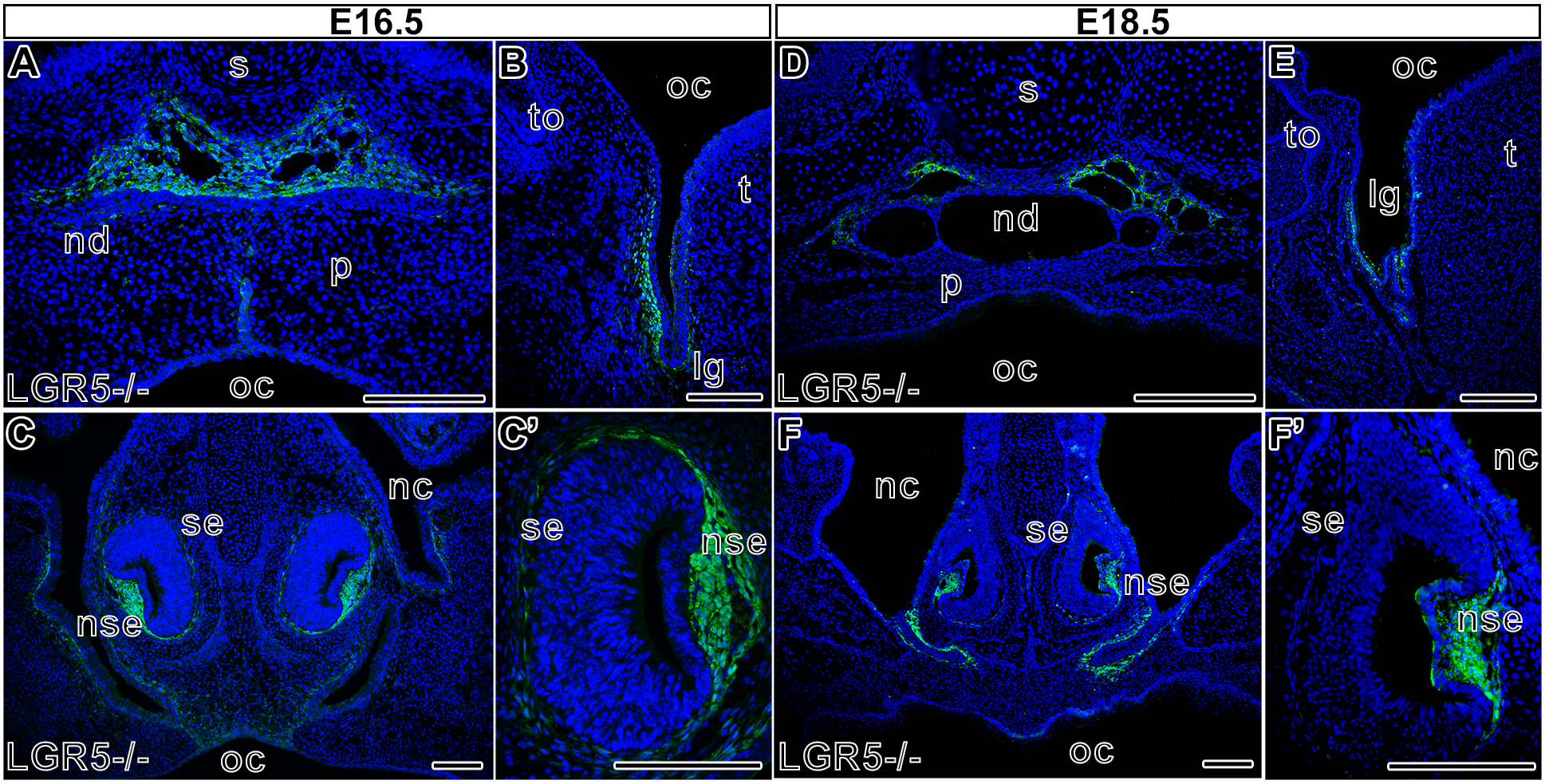

Supplement: Supplementary file 8 [file Image7.tif]

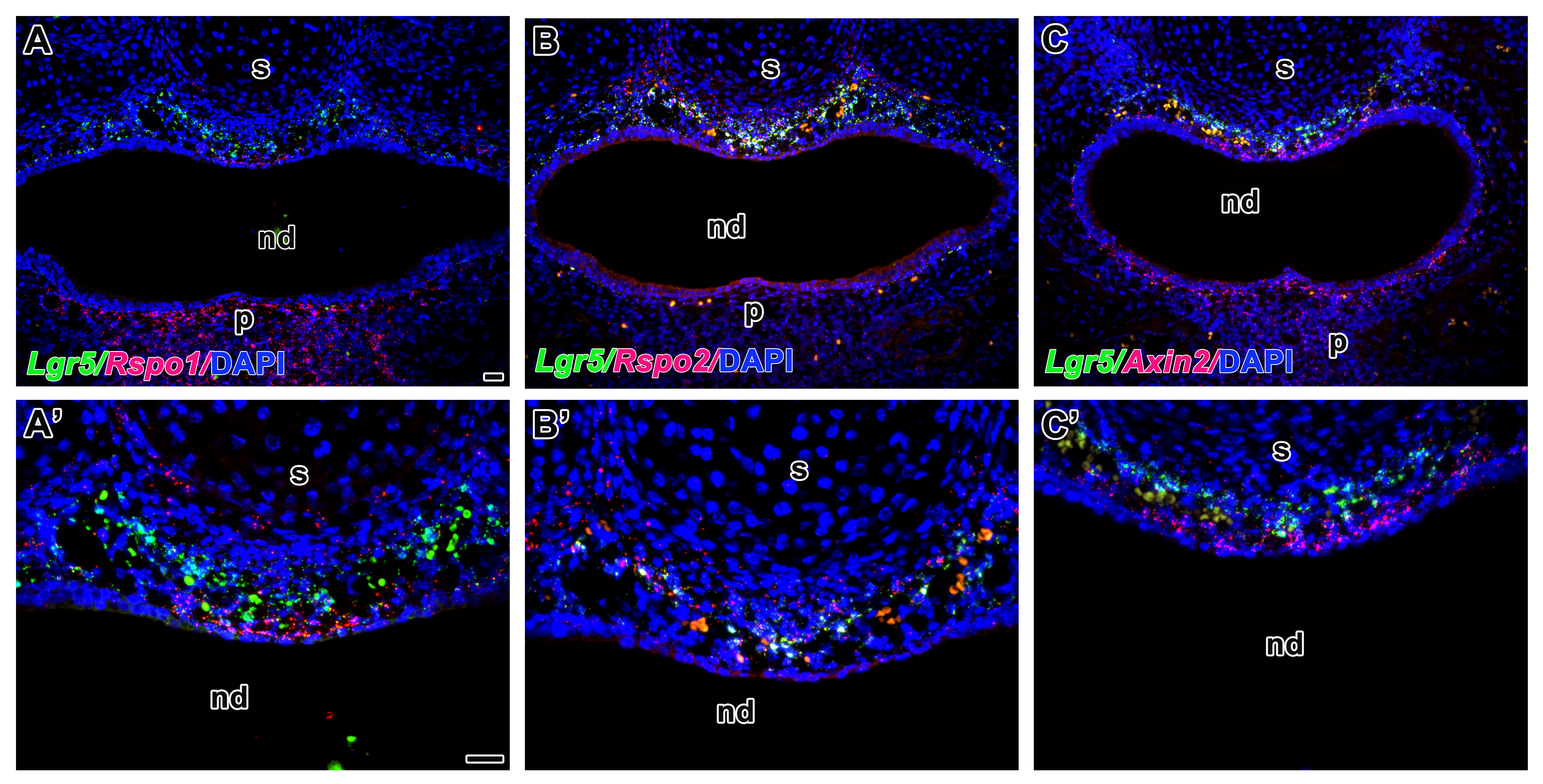

Supplement: Supplementary file 9 [file Image8.TIF]

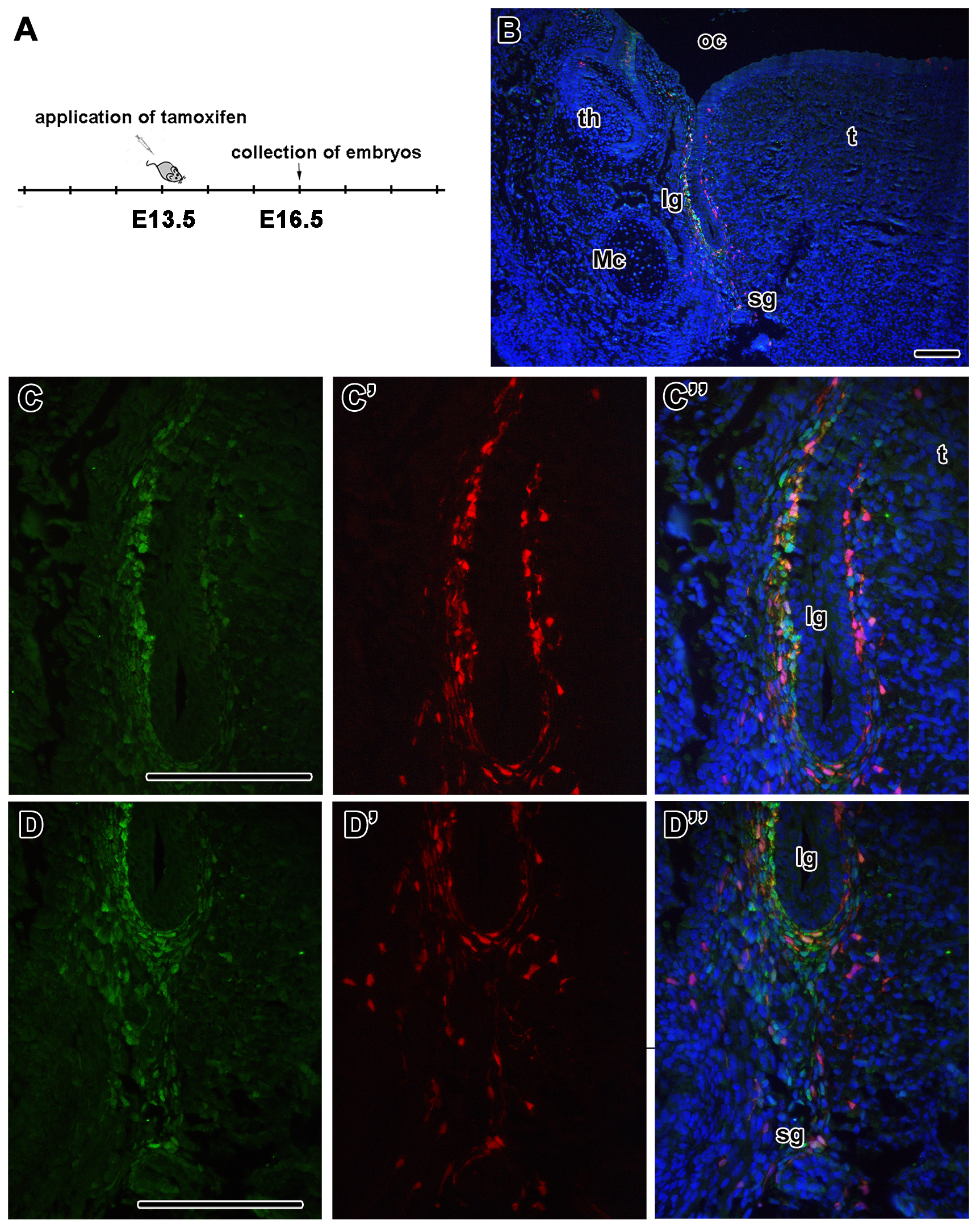

Supplement: Supplementary file 10 [file Image5.TIF]
